# Extracellular Vesicles in Cancer Drug Resistance: Roles, Mechanisms, and Implications

**DOI:** 10.1002/advs.202201609

**Published:** 2022-10-17

**Authors:** Qiurong Yang, Jing Xu, Jianmei Gu, Hui Shi, Jiayin Zhang, Jianye Zhang, Zhe‐Sheng Chen, Xinjian Fang, Taofeng Zhu, Xu Zhang

**Affiliations:** ^1^ Jiangsu Key Laboratory of Medical Science and Laboratory Medicine School of Medicine Jiangsu University Zhenjiang Jiangsu 212013 China; ^2^ Departmemt of Clinical Laboratory Medicine Nantong Tumor Hospital Nantong Jiangsu 226361 China; ^3^ Guangdong Provincial Key Laboratory of Molecular Target and Clinical Pharmacology School of Pharmaceutical Sciences and the Fifth Affiliated Hospital Guangzhou Medical University Guangzhou Guangdong 511436 China; ^4^ College of Pharmacy and Health Sciences St. John's University Queens NY 11439 USA; ^5^ Department of Oncology Lianyungang Hospital Affiliated to Jiangsu University Lianyungang Jiangsu 222000 China; ^6^ Department of Pulmonary and Critical Care Medicine Yixing Hospital affiliated to Jiangsu University Yixing Jiangsu 214200 China

**Keywords:** cancer therapy, drug delivery, drug resistance, extracellular vesicles, nanomedicine

## Abstract

Extracellular vesicles (EVs) are cell‐derived nanosized vesicles that mediate cell‐to‐cell communication via transporting bioactive molecules and thus are critically involved in various physiological and pathological conditions. EVs contribute to different aspects of cancer progression, such as cancer growth, angiogenesis, metastasis, immune evasion, and drug resistance. EVs induce the resistance of cancer cells to chemotherapy, radiotherapy, targeted therapy, antiangiogenesis therapy, and immunotherapy by transferring specific cargos that affect drug efflux and regulate signaling pathways associated with epithelial‐mesenchymal transition, autophagy, metabolism, and cancer stemness. In addition, EVs modulate the reciprocal interaction between cancer cells and noncancer cells in the tumor microenvironment (TME) to develop therapy resistance. EVs are detectable in many biofluids of cancer patients, and thus are regarded as novel biomarkers for monitoring therapy response and predicting prognosis. Moreover, EVs are suggested as promising targets and engineered as nanovehicles to deliver drugs for overcoming drug resistance in cancer therapy. In this review, the biological roles of EVs and their mechanisms of action in cancer drug resistance are summarized. The preclinical studies on using EVs in monitoring and overcoming cancer drug resistance are also discussed.

## Graphical abstract

Extracellular vesicles (EVs) mediate the resistance of cancer cells to chemotherapy, radiotherapy, targeted therapy, antiangiogenesis therapy, and immunotherapy. This review summarizes the mechanisms for the biological roles of EVs in cancer drug resistance, and discusses about the preclinical studies on using EVs in monitoring cancer therapy response and overcoming drug resistance.

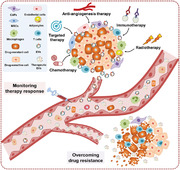

## 1 Introduction

Extracellular vesicles (EVs) are membrane‐bound, lipid bilayer‐enclosed, and nanosized vesicles produced by many types of cells.^[^ ^1^ ^]^ EVs are present in various human biofluids such as blood, urine, ascites, saliva, cerebrospinal fluid, bronchoalveolar lavage fluid, etc.^[^ ^2^ ^]^ There are three main subtypes of EVs: exosomes, microvesicles (MVs) or microparticles (MPs), and apoptotic bodies according to their origin and size. Exosomes are produced by the fusion of multivesicular bodies (MVBs) that contain intraluminal vesicles (ILVs) with plasma membrane followed by release into the extracellular area.^[^ ^3^ ^]^ MVs are formed by outward budding and fission of plasma membrane and released into the extracellular space.^[^ ^4^ ^]^ Apoptotic bodies are the largest subpopulation of EVs released by cells undergoing apoptosis.^[^ ^5^ ^]^ EVs carry a variety of bioactive cargos and transfer them from donor cells to recipient cells via distinct ways,^[^ ^6^ ^]^ which therefore has been described as a new mode of intercellular communication.

In recent years, cancer therapy has been greatly improved, whereas therapy failure caused by drug resistance remains a major challenge. Most cancer patients initially respond to drug therapy, but gradually develop resistance during treatment, which has become one of the main causes of cancer‐related death.^[^ ^7^ ^]^ Drug resistance is regulated by many intrinsic and extrinsic factors, such as genetic and phenotypic changes in cancer cells or microenvironmental cells, which may prevent drug uptake and promote drug efflux, thereby reducing drug effectiveness.^[^ ^8^ ^]^ Drug resistance could arise from horizontal transfer in which the resistance is disseminated from resistant cells to sensitive ones. For example, the acquisition of functional P‐glycoprotein (P‐gp/ABCB1) from resistant cells endows sensitive counterparts a persisted resistant phenotype.^[^ ^9^ ^]^

EVs were originally thought of as garbage bags for releasing cellular waste. The past two decades have witnessed extensive studies on the biology and function of EVs in cancer. The role of EVs in cancer drug resistance has been gradually recognized. EV‐mediated drug resistance has been reported in many therapies, such as chemotherapy, radiotherapy, targeted therapy, immunotherapy, and antiangiogenesis therapy. A wide spectrum of mechanisms has been reported to be responsible for EV‐mediated cancer drug resistance. EVs carry drug efflux pumps that are involved in the sequestration of anticancer drugs, which decreases drug concentration in cancer cells.^[^ ^9^, ^10^ ^]^ In response to chemotherapy, some of these drug efflux pumps are enriched in EVs and shuttled between cells, representing a common mechanism for transmitting drug resistance. EVs could also act as a bait to capture monoclonal antibodies targeting cancer‐associated ligands or receptors. In addition, EVs from drug‐resistant cancer cells transfer nucleic acids (DNA, mRNA, miRNA, lncRNA, and circRNA) and proteins to sensitive cells to promote epithelial‐mesenchymal transition (EMT), autophagy, metabolism, and cancer stemness, all of which are associated with the acquisition of drug resistance.^[^ ^11^ ^]^ Moreover, cancer cells actively interact with noncancer cells in the TME via EVs, which ultimately leads to therapy resistance and cancer progression. Therefore, a better understanding of the mechanism for EV‐mediated drug resistance has become an emerging area of research.

The specific cargos in EVs from different biofluids of cancer patients have been used as biomarkers for monitoring therapy response. Furthermore, EVs have been utilized as a target to reverse cancer drug resistance, for example, by antagonizing EV‐mediated signaling pathways that control drug resistance. Due to their unique biological properties, EVs have also been used as a novel delivery vehicle to overcome drug resistance. Herein, we reviewed the biological roles of EVs in the emergence of cancer drug resistance and the underlying molecular mechanisms responsible for their functions. We also highlighted the recent advances in the applications of EVs for monitoring cancer therapy response and overcoming cancer drug resistance. We expect that further study of EVs will shed lights on cancer liquid biopsy and offer new opportunities for improving cancer therapy.

## 2 The Biology of EVs

EVs were originally used to describe the lipid‐rich particles produced by platelets during blood coagulation.^[^ ^12^ ^]^ Later, EVs are referred to vesicles of unknown origin released from various cultured cells carrying 5ʹ‐nucleotidase activity.^[^ ^13^ ^]^ In 1987, Johnstone et al. first confirmed that the lipid‐rich membrane vesicles that expressed the transferrin receptor were produced during the maturation of reticulocytes to erythrocytes.^[^ ^14^ ^]^ One decade later, the researchers discovered that B lymphocytes and dendritic cells (DCs) could release similar endosome‐derived vesicles with potential antigen presentation and immune regulation functions.^[^ ^15^ ^]^ In 2007, Valadi et al. reported that EVs were capable of shuttling RNAs (mRNAs and miRNAs) between mast cells.^[^ ^16^ ^]^ The nomenclature “extracellular vesicles (EVs)” was proposed to define all lipid bilayer membrane‐enclosed vesicles in 2011.^[^ ^17^ ^]^ Overall, EVs can be divided into three categories according to their origin and size: apoptotic bodies (1000–5000 nm), microvesicles (also called ectosomes, 100–1000 nm), and exosomes (40–160 nm).^[^ ^18^ ^]^

The biogenesis of exosomes is a complex process which includes endocytosis, MVBs formation and exosome release. MVBs, formed by double invagination of the plasma membrane, are characterized by containing several ILVs. MVBs can be localized to lysosomes for degradation or fuse with the plasma membrane to release into extracellular space.^[^ ^3^ ^]^ Both endosomal sorting complex required for transport (ESCRT)‐dependent and ‐independent mechanisms have been reported in MVB formation.^[^ ^19^ ^]^ In addition, autophagy‐related genes (Atg) can promote the biogenesis and release of MVBs. ISGylation, a new ubiquitin‐like modifier, regulates exosome production and ISGylation of the MVB protein TSG101 induces its aggregation and degradation, thereby inhibiting exosome secretion. MVs are produced by an outward budding and fission process from plasma membrane, and are subsequently released into extracellular space.^[^ ^20^ ^]^ Apoptotic bodies contain nuclear and cytoplasmic contents surrounded by membranes formed during apoptosis. EVs contain many biologically active substances, including lipids, proteins, nucleic acids (DNA or RNA), metabolites, and others. EVs can act in an autocrine or paracrine mode to affect their derived cells or adjacent and distant cells.^[^ ^21^ ^]^ EVs enter into recipient cells through three main ways: direct fusion with plasma membrane, receptor–ligand interaction, and fusion with inner membrane after endocytosis.^[^ ^22^ ^]^

The processes of EV biogenesis, release, and uptake are strictly and dynamically regulated. Milman et al. suggest that Rab guanosine triphosphatases are critically involved in the intracellular trafficking steps and the soluble N‐ethylmaleimide‐sensitive fusion attachment protein receptor (SNAREs) regulate EV secretion pathways.^[^ ^21^, ^23^ ^]^ Hsu et al. show that Rab35 depletion results in endosomal vesicle accumulation and exosome release inhibition, indicating that Rab family proteins are important regulators of EV biogenesis.^[^ ^24^ ^]^ Furthermore, EV release and uptake increase at low pH, and more caveolin‐1 protein are transmitted by exosomes under acidic conditions.^[^ ^25^ ^]^ Hypoxia promotes exosome release from cancer cells.^[^ ^26^ ^]^ Increased external pressure also promotes exosome release. For instance, the patients with hypertension are found to have higher levels of circulating EVs than healthy controls.^[^ ^27^ ^]^

Increasing evidence suggests that EVs play a vital role in normal physiology and disease pathology. For instance, EVs remove unnecessary components from the cells, mediate specific intercellular information exchange and communication, and activate signaling pathways in cells.^[^ ^28^ ^]^ EVs act as a homeostatic integrator in physiological and dynamic balance as well as disease development and procession by participating in the communication between cells.^[^ ^29^ ^]^ Olefsky and co‐workers demonstrate that exosomes secreted by M1 macrophages contain miRNA‐155, which can target adipocytes and render them resistant to insulin; however, exosomes secreted by M2 macrophages containing miR‐223 which can also target adipocytes but make them more sensitive to insulin.^[^ ^30^ ^]^ EVs also participate in multiple systemic pathological situations, such as blood coagulation, immune responses, infectious diseases, metabolic diseases, central nervous system‐related diseases, musculoskeletal diseases, and cancers.^[^ ^31^ ^]^

## 3 EVs in Cancer Biology

Cancer have several hallmarks, such as continuous cell proliferation, evasion of growth inhibition and cell death, induction of angiogenesis, invasion and metastasis.^[^ ^32^ ^]^ Previous studies suggest that EVs are involved in almost all aspects of cancers.^[^ ^22^ ^]^ It has been reported that different sources of EVs could directly activate signaling pathways that maintain cancer cell proliferation through their specific cargos.^[^ ^33^ ^]^ Cancer metastasis is a multistep and multifactor process, which includes separation from the primary cancer, invasion of cancer cells into the basement membrane, invasion of blood vessels and lymphatics, and colonization in target organs. Intriguingly, EVs participate in the metastatic process of cancer cells through many ways, such as promotion of cancer cell migration and invasion, establishment of premetastatic niche, and remodeling of extracellular matrix.^[^ ^34^ ^]^ Cancer stem cells (CSCs) are a subset of cancer cells with self‐renewal and differentiation potential, which is one of the leading causes for cancer therapy failure and recurrence. EVs participate in the dynamic maintenance of CSC population, providing a superior condition for cancer development.^[^ ^35^ ^]^ Angiogenesis is critical for cancer progression, and is dynamically regulated by many factors. EVs from distinct cells mediate signal transduction in vascular development, growth, and maturation as well as regulation of angiogenesis‐related gene expression.^[^ ^36^ ^]^ In addition, EVs regulate autophagy by transferring active substances and activating intracellular autophagy‐related signaling pathways in cancer cells.^[^ ^37^ ^]^ Abnormal metabolism is observed in cancer and fast‐growing cancer cells require more energy. Mounting evidence indicates that EVs regulate the metabolic state of recipient cells, which in turn, promotes cancer metastasis and drug resistance.^[^ ^38^ ^]^ TME is a highly heterogeneous cellular network consisting of cellular and noncellular components. EVs have been shown to promote the remodeling of the immunosuppressive microenvironment by reeducating immune cells from a cancer‐suppressing state to cancer‐promoting state.^[^ ^39^ ^]^ Moreover, EVs can mediate the transmission of genetic information related to drug resistance.^[^ ^9^, ^40^ ^]^ Previous studies have shown that EVs regulate the sensitivity of cancer cells to different therapies, including chemotherapy, radiotherapy, antiangiogenesis therapy, targeted therapy, and immunotherapy. Thus, a timely and in‐depth understanding of the role of EVs in cancer drug resistance will allow to develop more effective anticancer approaches.

## 4 EVs and Cancer Drug Resistance

**Figure 1:**
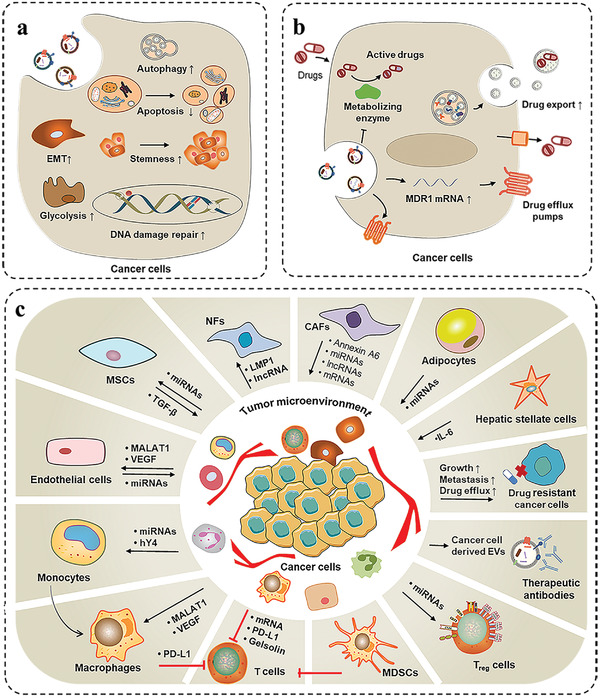
The role and mechanism of EVs in cancer drug resistance. a) EVs transfer specific cargos that regulate signaling pathways associated with autophagy, metabolism, EMT, and CSCs, thus play important roles in the development of cancer drug resistance. b) EVs contain drug efflux transporters that directly mediate the active extrusion of drugs, leading to the development of drug resistance. c) EVs transport biological active substances to mediate multiple layers of interactions between cancer cells and TME cells, leading to the acquisition of drug resistance.

Drug resistance is a main problem in cancer therapy. The resistance can be inherent or acquired after initial response to treatment. Accumulating evidence indicates that EVs play important roles in cancer drug resistance (**Figure** **1**). EVs can transfer specific cargos including drug resistance‐related proteins, nucleic acids, and metabolites to cancer cells or directly package and sequester drugs out of the cells, leading to the development of drug resistance (**Table** **1**).

**Table 1:** Different cargos in EV‐mediated cancer drug resistance

Cargo type	Molecules	Targets	Drug	Outcome	Refs.
Drug efflux pumps	Glycoprotein (P‐gp)	–	Daunorubicin	Promote drug efflux	[9]
		–	Doxorubicin	Induce a doxorubicin‐resistant phenotype	[43]
		–	Docetaxel	Promote drug resistance transfer	[45]
	ABCG2	–	Mitoxantrone	Promote drug efflux	[46]
		PI3K‐Akt signaling pathway	mitoxantrone and topotecan	Confer multidrug resistance	[48]
	ABCA3	Modulate exosome biogenesis	Therapeutic anti‐CD20 antibodies	Reduce susceptibility of target cells to antibody‐mediated lysis	[10a]
	–	GEM increases the expression of efflux and influx proteins	Gemcitabine (GEM)	Promote drug efflux	[49]
	–	Sorting drug at the surface of tumor EVs	Bevacizumab	Promote drug efflux	[50]
	Rab27B	–	5‐Fluorouracil (5‐FU)	Promote drug efflux	[51]
	ATP‐binding cassette subfamily B member 1 (ABCB1)	Rab8B and Rab5	Doxorubicin	Develop resistant phenotype	[52]
	ATP1B3	–	Cisplatin	Promote cisplatin resistance	[53]
Proteins	TrpC5	P‐glycoprotein	Doxorubicin	Stimulate P‐gp production and confer chemoresistance	[54]
	p‐STAT3	–	5‐FU	Contribute to acquired 5‐FU resistance in CRCs	[55]
	Acid sphingomyelinase	PARP and caspase 3 pathways	Melphalan and bortezomib	Induce chemoresistance	[57]
	Death receptor 5	–	Tumor necrosis factor‐related apoptosis inducing ligand (TRAIL)	Decreases TRAIL sensitivity of colon cancer cells	[56]
	PTPRZ1–MET fusion	–	Temozolomide	Transmit chemoresistance	[58]
	RBM11	MDM4 and Cyclin D1	Temozolomide, cisplatin and *γ*‐radiation	Switch splicing of MDM4 and Cyclin D1 toward the expression of more oncogenic isoforms	[59]
	Gelsolin	CD8 ^+^ T cell function and glutathione production	Cisplatin	Promote the overall survival of ovarian cancer cells; inhibit CD8^+^ T cell function and enhance chemoresistance	[60, 61]
	PDGFR*β*	Activation of PI3K/Akt signaling	BRAF inhibitor	Escape from BRAF inhibition and transmit resistance	[62]
	Latent membrane protein 1	P38 MAPK signaling	–	Induce recipient cell proliferation and invasion, suppress apoptosis, and thus promote radioresistance	[63]
	Wnt	Wnt signaling	5‐FU and oxaplatin	Induce the dedifferentiation of cancer cells to promote chemoresistance	[175]
	EphA2	–	Gemcitabine	Transmit chemoresistance	[64]
	Neuromedin U	HSP27	Lapatinib, trastuzumab, neratinib, and afatinib	Stabilize HER2 protein levels and promote drug resistance.	[65]
		TGF*β*1 and PD‐L1	Trastuzumab	Enhance resistance to antitumor immune response	[66]
	PD‐L1	–	Anti‐PD‐1 antibody	Suppress the function of CD8 T cells and facilitate tumor growth	[68, 69]
	PKM2	–	Cisplatin	Transmit cisplatin resistance to sensitive NSCLC cells	[176]
	Wild type EGFR	Rab GTPase (RAB17)	Osimertinib	Promote osimertinib resistance in NSCLC	[70]
	Yes‐associated protein 1 (YAP1)	Chicken ovalbumin upstream promoter transcription factor 2 (COUP‐TFII)	Enzalutamide	Induce the abilities of cancer stemness, lipid metabolism and enzalutamide resistance in the parental cells	[71]
	Carbonic anhydrase 1 (CA1)	NF‐*κ*B and STAT3 pathways	Cyclophosphamide, doxorubicin, vincristine, prednisone, and rituximab	Promote chemotherapy resistance in diffuse large B cell lymphoma	[72]
		–	PTX; ERK inhibitor and chloroquine	Promote cancer cell survival and therapy resistance	[73]
	MIF (migration inhibitory factor)	TIMP3/PI3K/Akt axis	Temozolomide	Enhance temozolomide resistance in glioma	[74]
	Annexin A6 (ANXA6)	EGFR	Gemcitabine	Inhibit ubiquitination and degradation of EGFR and induce gemcitabine resistance	[75]
	UCH‐L1 and P‐gp	–	Doxorubicin	Transfer chemoresistance	[76]
	CD44	–	Doxorubicin	Enhance cell proliferation and reduce drug sensitivity	[77]
	DNMT1	–	Cisplatin	Render host cell the resistance to cytotoxicity of cisplatin	[90]
DNA	Mitochondrial DNA	Estrogen receptor‐independent oxidative phosphorylation (OXPHOS)	Hormone	Promote an exit from dormancy of therapy‐induced cancer stem‐like cells and lead to endocrine therapy resistance	[79]
mRNAs	ΔNp73	–	Oxaliplatin	Promote cell proliferation and confer drug resistance on the recipient cells	[81]
	Glycerol kinase 5	SREBP1/SCD1 signaling pathway	Gefitinib	Inhibit mitochondrial damage, caspase activation, cell cycle arrest, and apoptosis	[82]
	Truncated ALK	MAPK signaling pathway	BRAF inhibitor	Inhibit cell apoptosis and promote drug resistance	[83]
	VEGF and VEGF receptor (VEGFR)	VEGFR/glycolysis pathway	Ara‐C	Induce glycolysis in HUVECs and lead to vascular remodeling and acquisition of chemoresistance	[85]
	MGMT	–	Temozolomide	Inhibit TMZ‐induced apoptosis	[86]
	ZEB1	–	Cisplatin and gemcitabine	Induce a mesenchymal phenotype in recipient cells	[84]
	PSMA3 and PSMA3‐AS1	PSMA3‐AS1/PSMA3 signaling pathway	Proteasome inhibitors (PI)	Transmit PI resistance from MSCs to MM cells	[87]
	MET	–	Icotinib	Mediate the migration and invasion of NSCLC cells	[88]
	N6‐methyladenosine RNA demethylase FTO	–	Gefitinib	Increase ABCC10 of recipient cells in a m^6^A‐dependent manner and promote gefitinib resistance	[89]
	DNMT1	–	Cisplatin	Render host cell resistance to cytotoxicity of cisplatin	[90]
miRNAs	miR‐155	TERF1	Cisplatin	Mediate crosstalk between NBL cells and human monocytes	[92]
		–	Cisplatin	Induce EMT, migratory potential, and resistant phenotype	[93]
		DCK	Gemcitabine	Suppress key gemcitabine‐metabolizing enzyme DCK and confer chemoresistance	[102]
	miR‐9‐5p, miR‐195‐5p, and miR‐203a‐3p	ONECUT2	Docetaxel and doxorubicin	Promote breast cancer stemness	[94]
	miR‐194‐5p	E2F3	Radiotherapy	Enhance DNA damage response in tumor repopulating cells to potentiate tumor repopulation	[98]
	miR‐603	IGF1 and IGF1R	Radiotherapy	Promote the CSC state and upregulate DNA repair to promote acquired resistance	[95]
	miR‐208a	p21	Radiotherapy	Promote cell proliferation and induce radioresistance	[96]
	miR‐340‐5p	KLF10/UVRAG	Radiotherapy	Induce radioresistance	[97]
	miR‐30b‐3p	RHOB	Temozolomide	Result in decreased apoptosis and increased proliferation, thus promote TMZ resistance in the recipient cells	[99]
	miR‐210	mTOR signaling pathway	Gemcitabine	Activate the mammalian target of rapamycin (mTOR) signaling pathway	[100]
	miR‐500a‐3p	FBXW7	Cisplatin	Enhance stemness properties and resistance	[101]
	miR‐31‐5p	MutL homolog 1 (MLH1)	Sorafenib	Downregulate MLH1 expression and thus promote sorafenib resistance	[103]
	miR‐222‐3p	SOCS3	Gemcitabine	Enhance the proliferation, gemcitabine resistance, migration, invasion, and antianoikis of sensitive cells	[104]
	miR‐761	TRIP6, LMNA, SIRT3	Pazopanib	Confer increased resistance	[105]
	miR‐46146	PDCD10	Oxaliplatin	Contribute to the chemoresistance transfer	[106]
	miR‐21	PDCD4	5‐FU	Downregulate TPM1 and PTEN; promote proliferation and invasion	[107b]
		PTEN and PDCD4	Cisplatin	Enhance the chemoresistance of cancer cells and decrease the DNA damage signaling in response to cisplatin	[107a]
	miR‐3648 and miR‐522‐3p	PI3K/Akt signaling pathway	Gefitinib	Induce EGFR‐TKI resistance	[108]
	miR‐425‐3p	AKT1	Cisplatin	Activate autophagy to confer cisplatin resistance in NSCLC	[109]
	miR‐425‐3p	–	Cisplatin	Alter the sensitivity of breast cancer cells to DDP	[110]
	miR‐214	–	Gefitinib	Transfer chemoresistance	[111a]
	miR‐100‐5p	mTOR signaling pathway	Cisplatin	Transfer chemoresistance	[111b]
	miR‐21‐5p and miR‐486‐3p	–	ALK‐tyrosine kinase inhibitor crizotinib or ceritinib	Increase drug resistance of sensitive subclones	[112]
	miR‐769‐5p	CASP9	Cisplatin	Confer cisplatin resistance	[113]
	miR‐501	BLID	Doxorubicin	Downregulate BLID, and thus Inactivate caspase‐9/‐3 and attenuate apoptosis; increase proliferation, migration, and invasion and attenuate apoptosis	[114a]
	miR‑155‑5p	GATA3 and TP53INP1	PTX	Induce EMT and chemoresistant phenotypes	[114b]
	miR‐1238	CAV1/EGFR pathway	Temozolomide	Confer chemoresistance	[115]
	miR‐32‐5p	PTEN/PI3K/Akt pathway	5‐FU	Induce multidrug resistance via promoting angiogenesis and EMT	[116]
	miR‐19b and miR‐20a	–	Daunorubicin	Transfer resistance from chemoresistant cells to sensitive cells	[117]
	miR‐21‐5p	Glycolysis, ATP‐binding cassette family, and a detoxification enzyme	Carboplatin	Activate glycolysis and increase the expression of ATP‐binding cassette family and a detoxification enzyme	[221]
	miR‐21‐3p and miR‐891‐5p	DNA repair mechanisms	Carboplatin	Increase the expression of proteins involved in DNA repair	[221]
lncRNAs	lncARSR	miR‐34/miR‐449	Sunitinib	Competitively bind miR‐34/miR‐449 to facilitate AXL and c‐MET expression	[120]
	PART1	miR‐129/Bcl‐2 axis	Gefitinib	Promote gefitinib resistance	[121]
	SBF2‐AS1	miR‐151a‐3p	Temozolomide	Increase X‐ray repair cross complementing 4 (XRCC4) expression and thus enhance DNA double‐stand break repair	[122]
	UCA1	miR‐143/FOSL2 axis	Cisplatin	Modulate cisplatin resistance through the miR‐143/FOSL2 pathway in ovarian cancer	[123]
		–	Tamoxifen	Increase cell viability and decrease tamoxifen‐induced apoptosis	[124]
	HOTTIP	miR‐218/HMGA1 axis	Cisplatin	Promote cisplatin resistance via activating HMGA1	[125]
	Linc‐VLDLR	ABCG2	Sorafenib, camptothecin, and doxorubicin	Promote cell viability and cell cycle progression	[126]
		ABCG2	Doxorubicin	Increase the expression of ABCG2 and thus regulate drug resistance	[127]
	Linc‐ROR	–	Sorafenib, doxorubicin, and camptothecin	Reduce chemotherapy‐induced cell death	[128]
	AGAP2‐AS1	–	Trastuzumab	Inhibit trastuzumab‐induced cell cytotoxicity	[129a]
		ATG10	Trastuzumab	Regulate trastuzumab resistance via inducing autophagy in breast cancer cells	[129b]
	RP11 838N2.4	–	Erlotinib	Promote erlotinib resistance in NSCLC	[130]
	lnc‐SOX2	miR‐627‐3p/Smads signaling pathway	EGFR‐TKI	Enhance EGFR‐TKI resistance	[134]
	lnc‐TALC	–	Temozolomide	Enhance the repair of DNA damage induced by temozolomide and lead to resistance	[135]
	H19	–	Gefitinib	Reduce gefitinib‐induced cell cytotoxicity	[131]
		miR‐615‐3p/ATG7 axis	Erlotinib	Facilitate erlotinib resistance	[132]
		–	Doxorubicin	Promote cell viability, colony‐forming ability, and reduce apoptosis	[133]
	HIF‐1*α*‐stabilizing long noncoding RNA (HISLA)	HIF‐1*α*	Docetaxel	Block the interaction of PHD2 and HIF‐1*α* to inhibit the hydroxylation and degradation of HIF‐1*α*	[185]
circRNAs	circ_0000338	–	5‐FU and oxaliplatin	Promote cancer progression in chemoresistant CRCs	[91c]
	circUHRF1	miR‐449c‐5p/TIM‐3 axis	Anti‐PD1 immunotherapy	Inhibit NK cell function (inhibit NK cell‐derived IFN‐*γ* and TNF‐*α* secretion) by upregulating the expression of TIM‐3 via inhibition of miR‐449c‐5p	[136]
	circ‐CPA4	let‐7 miRNA/PD‐L1 axis	Cisplatin	Downregulate let‐7 miRNA to upregulate intracellular and extracellular PD‐L1 in NSCLC cells	[137]
	circUSP7	miR‐934/SHP2 axis	Anti‐PD1 immunotherapy	Induce CD8^+^ T cell dysfunction and anti‐PD1 resistance	[138]
	ciRS‐122	miR‐122/PKM2 axis	Oxaliplatin	Promote glycolysis and drug resistance through miR‐122 sponging and PKM2 upregulation	[139]
	Circ_UBE2D2	miR‐200a‐3p	Tamoxifen	Regulate cell viability, metastasis, and the level of ER*α*	[141]
	hsa_circ_0002130	miR‐498	Osimertinib	Target miR‐498 to regulate glycolysis	[140]
	circRNA‐SORE	YBX1	Sorafenib	Prevent YBX1 nuclear interaction with the E3 ubiquitin ligase PRP19 and thus blocks PRP19‐mediated YBX1 degradation	[142]
	circ_0000337	miR‐377‐3p/JAK2 axis	Cisplatin	Accelerate drug resistance, cell growth, and metastasis of sensitive cells	[143]
	hsa_circ_0014235	miR‐520a‐5p/CDK4 axis	Cisplatin	Promote NSCLC cell resistance to cisplatin and malignant development	[144]
	circFoxp1	miR‐22 and miR‐150‐3p	Cisplatin	Promote cell proliferation and confer DDP resistance	[145]
	Cdr1as	miR‐1270/Suppressor of Cancer Cell Invasion (SCAI) axis	Cisplatin	Inhibit cell proliferation, promote the cisplatin‐induced cell apoptosis and sensitize ovarian cancer to cisplatin	[146]
	circZNF91	miR‐23b‐3p	Gemcitabine	Lead to glycolysis and GEM chemoresistance of recipient cells	[147]

### 4.1 Drug Efflux Pump

Multidrug resistance (MDR) is a major obstacle for cancer therapy, most of which are related to the increased expression of drug efflux pumps.^[^ ^41^ ^]^ Increasing studies suggest that EVs contain a variety of drug efflux pumps, such as transporters of the ATP‐binding cassette (ABC) superfamily including P‐glycoprotein (P‐gp/ABCB1/MDR1), ABC transporter G2 (ABCG2/BCRP), and multidrug‐resistant protein 1 (MRP1/ABCC1), which directly mediate the active extrusion of drugs. In addition, previous studies suggest that EVs can transfer drug‐efflux pumps from drug‐resistant cancer cells to drug‐sensitive cancer cells to acquire drug resistance.^[^ ^9^, ^42^ ^]^ For instance, osteosarcoma MDR cells are able to spread their doxorubicin (DOX)‐resistant trait to sensitive cells by exosomes that carry *MDR‐1* mRNA and its product P‐gp.^[^ ^43^ ^]^ Paclitaxel (PTX)‐resistant human ovarian cancer cells derived MVs transmit functional P‐gp to chemosensitive wild‐type parental cells and enhance their chemoresistance.^[^ ^44^ ^]^ EVs also transmit drug resistance from docetaxel‐resistant breast cancer cells to their sensitive counterparts by transferring P‐gp/ABCB1.^[^ ^45^ ^]^ Bebawy et al. suggest that MPs from drug‐resistant cancer cells (VLB100) effectively transfer functional P‐gp/ABCB1 to drug‐sensitive cells (CCRF‐CEM).^[^ ^9^ ^]^ Ifergan et al. suggest that EVs mediate ABCG2‐dependent drug sequestration and resistance. ABCG2 is highly expressed at cell–cell attachment zones between neighboring cancer cells, which comprises the membrane of EVs and rapidly sequesters the anticancer drug mitoxantrone.^[^ ^46^ ^]^ They also suggest that drug concentration within ABCG2‐rich EVs may be used as a sensitive functional marker to quantify MDR levels in malignant cells.^[^ ^47^ ^]^ Other researchers have explored the signaling pathways that regulate ABCG2/BCRP accumulation in EVs and suggest that inhibition of Akt signaling may lead to a gradual relocalization of ABCG2 from EV membrane to cytoplasm, inhibiting EV production and reversing MDR.^[^ ^48^ ^]^ Muralidharan‐Chari et al. suggest that the expression of efflux and influx proteins is increased in MVs after gemcitabine (GEM) treatment.^[^ ^49^ ^]^ The amount of released MVs is correlated with the ability of pancreatic cancer cells to resist GEM and the inhibitor of MV release increases cancer cell sensitivity to GEM.

Invasive B cell lymphoma cells are found to produce and release exosomes to protect target cells from antibody attack. The lysosome‐associated ABC transporter A3 (ABCA3) regulates exosome biogenesis and ABCA3 depletion enhances the sensitivity of target cells to antibody‐mediated cytotoxicity.^[^ ^10a^ ^]^ Similarly, bevacizumab is captured by glioblastoma (GBM) cells derived EVs. Inhibition of EV production increases the effect of bevacizumab on GBM cell viability, suggesting a specific antibody shedding mechanism for therapeutic resistance and combination of bevacizumab with a local blockade of EV‐dependent intercellular communication may improve treatment efficacy.^[^ ^50^ ^]^

Recent studies suggest that transient exposure to some chemotherapeutic drugs stimulates EV release and recycling, promoting drug resistance in cancer cells. For instance, Rab27B is highly expressed in 5‐fluorouracil (FU)‐resistant liver cancer cells and these cells secrete more exosomes upon 5‐FU stimulation, whereas knockdown of Rab27B decreases the number of exosomes and increases the intracellular concentration of 5‐FU.^[^ ^51^ ^]^ Chemotherapeutic drug exposure increases Rab8B‐mediated release of ABCB1‐containing EVs from drug‐resistant cells and promotes the recycling of these EVs to the drug‐sensitive cells.^[^ ^52^ ^]^ Khoo et al. reveal that a low level of cisplatin accumulates in drug‐resistant oral squamous cell carcinoma (OSCC) cells, but a high level of cisplatin is detected in their EVs. A proton pump inhibitor inhibits the release of EVs, thus increasing OSCC drug sensitivity in the resistant cells.^[^ ^53^ ^]^

### 4.2 EV Proteins

In addition to drug efflux pumps, EVs also contain other functional proteins that regulate the acquisition of drug resistance in cancer cells. For instance, Ma et al. demonstrate that transient receptor potential channel 5 (TrpC5), a receptor potential protein with Ca^2+^ permeability, is upregulated in DOX‐resistant breast cancer cells and accumulates in their secreted EVs. EV‐mediated intercellular transfer of TrpC5 allows the recipient cells to acquire Ca^2+^‐permeable channel, stimulating P‐gp/ABCB1 production and thus endowing nonresistant cells with chemoresistance.^[^ ^54^ ^]^ Zhang et al. suggest that exosomal transfer of phosphorylated STAT3 (signal transducer and activator of transcription 3) contributes to the acquisition of 5‐FU resistance in colorectal cancer cells (CRCs).^[^ ^55^ ^]^ Moreover, CRCs secrete death receptor 5‐contained EVs to decrease their sensitivity to tumor necrosis factor‐related apoptosis inducing ligand (TRAIL).^[^ ^56^ ^]^ Acid sphingolipase (ASM) is reported to be upregulated in primary multiple myeloma (MM) cells and ASM‐containing exosomes are able to transfer the drug‐resistant phenotype to chemosensitive cells.^[^ ^57^ ^]^

Exosomes are carriers of cancer‐promoting factors and fusion genes that participate in GBM development and drug resistance. Exosomes that contain the PTPRZ1‐MET fusion (ZM fusion) gene could promote temozolomide (TMZ) resistance in GBM cells. In addition, ZM fusion network proteins in exosomes activate many oncogenic pathways in the recipient cells.^[^ ^58^ ^]^ Pavlyukov et al. demonstrate that apoptotic cell‐derived EVs promote therapy resistance of GBM cells via intercellular transfer of splicing factors such as RBM11, which is upregulated in GBM cells after therapy and is shed in EVs. After internalization by the recipient cells, RBM11 alters the splicing of MDM4 and Cyclin D1 and enables them to express more oncogenic isoforms.^[^ ^59^ ^]^ Exosomal plasma gelsolin (pGSN) is upregulated in an HIF1*α*‐dependent manner in cisplatin‐resistant ovarian cancer cells. pGSN has been shown to transform chemosensitive cells to resistant ones through both autocrine and paracrine mechanisms.^[^ ^60^ ^]^ The same group suggests that pGSN transported by exosomes inhibits the function of CD8^+^ T cells and regulates the production of glutathione, leading to chemoresistance in ovarian cancer.^[^ ^61^ ^]^ PDGFR*β*, a drug resistance driver, is transmitted by EVs to melanoma cells to activate the PI3K/Akt signaling pathway, thus helping them escape from the suppression by BRAF inhibitor.^[^ ^62^ ^]^ EVs from LMP1^+^ nasopharyngeal carcinoma (NPC) cells promote radioresistance by stimulating P38 MAPK signaling pathway.^[^ ^63^ ^]^ Fan et al. suggest that exosomes containing ephrin‐a receptor 2 (EphA2) confer GEM‐resistance in pancreatic cancer cells.^[^ ^64^ ^]^ The expression of neuromedin U (NmU) increases in HER2 targeted therapy‐resistant cells and overexpression of NmU in drug‐sensitive cells confers resistance to HER2‐targeting drugs.^[^ ^65^ ^]^ In addition, HER2^+^ breast cancer cells release EVs that overexpress NmU, which upregulates the expression of TGF*β*1 and PD‐L1, leading to the resistance to trastuzumab.^[^ ^66^ ^]^

**Figure 2:**
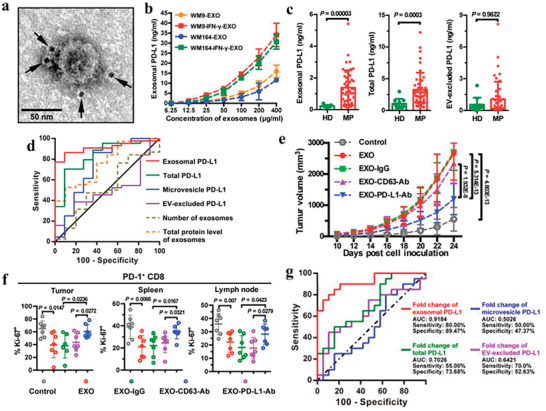
Exosomal PD‐L1 contributes to immunosuppression and is associated with anti‐PD‐1 response. a) A representative TEM image of WM9 cell‐derived exosomes immunogold‐labeled with anti‐PD‐L1 antibodies. Arrowheads indicate 5 nm gold particles. b) PD‐L1 concentration on the surface of exosomes isolated from indicated cell types. c) ELISA of circulating exosomal PD‐L, total PD‐L1 or EV‐excluded PD‐L1 in healthy donors (HD) and melanoma patients (MP). d) ROC curve analysis for the indicated parameters in patients with metastatic melanoma compared to healthy donors. e) Growth curve of PD‐L1 (KD) B16‐F10 tumors with indicated treatments. f) The proportions of Ki‐67^+^PD‐1^+^ CD8 TILs or splenic or lymph node CD8 T cells after indicated treatments. g) ROC curve analysis for the maximum fold change of circulating exosomal PD‐L1 at week 3–6 in clinical responders compared to nonresponders. Reproduced with permission.^[^ ^68^ ^]^ Copyright 2018, Springer Nature.

PD‐1/PD‐L1 antibodies have been proved to be effective agents for cancer therapy.^[^ ^67^ ^]^ However, anti‐PD‐1 therapy is restrained by low response rate and PD‐L1‐mediated immune evasion. Chen et al. show that metastatic melanoma cells release EVs that express PD‐L1, which could be enhanced by interferon‐*γ* (IFN‐*γ*), thus inhibiting CD8^+^ T cell function and promoting cancer cell proliferation. In patients who suffer from metastatic melanoma, circulating exosomal PD‐L1 levels have a positive association with IFN‐*γ* levels and change during anti‐PD‐1 therapy, implying that exosomal PD‐L1 may serve as a predictor of anti‐PD‐L1 therapy (**Figure** **2**).^[^ ^68^ ^]^ Guan et al. demonstrate that hepatocyte growth factor‐regulated tyrosine kinase substrate phosphorylation could induce the production of PD‐L1^+^ exosomes, thereby leading to resistance to anti‐PD‐1 treatment as a result of low infiltration of cytotoxic CD8^+^ T cells into tumors.^[^ ^69^ ^]^

Furthermore, osimertinib promotes exosome release via upregulating RAB17 and exosome‐mediated intercellular transfer of wild‐type EGFR protein to EGFR‐mutant cancer cells, thus activating downstream PI3K/Akt and MAPK signaling pathways and promoting osimertinib resistance.^[^ ^70^ ^]^ Lee et al. suggest that yes‐associated protein 1 (YAP1) is upregulated in prostate cancer cells with resistance to enzalutamide (EzaR), an androgen receptor inhibitor. EVs from EnzaR cells endow the sensitive cells resistance to enzalutamide, which is abolished when YAP1 is depleted by siRNA, suggesting that YAP in EVs is a key factor in the acquisition of enzalutamide resistance.^[^ ^71^ ^]^ Exosomal CA1 (carbonic anhydrase 1) level is higher in chemoresistant diffuse large B cell lymphoma (DLBCL) cells than its chemosensitive counterparts. Specifically, exosomal CA1 contributes to chemotherapy resistance by regulating NF‐*κ*B and STAT3 pathways.^[^ ^72^ ^]^ Moreover, exosomal survivin from pancreatic ductal adenocarcinoma (PDAC) cells strongly enhances the survival of PDAC cells upon serum starvation, and significantly compromises the effectiveness of PTX.^[^ ^73^ ^]^ Wei et al. show that migration inhibitory factor (MIF) is enriched in glioma cells with TMZ resistance and it transfers resistance to sensitive cells by inhibiting TIMP3 and activating the PI3K/Akt pathway.^[^ ^74^ ^]^ Li et al. suggest that annexin A6 (ANXA6) protein is enriched in exosomes derived from drug resistant cancer cells, which inhibit the ubiquitination and degradation of EGFR, thereby inducing GEM resistance in triple‐negative breast cancer.^[^ ^75^ ^]^ The DOX‐resistant breast cancer cells transfer exosomes that carry UCH‐L1 and P‐gp/ABCB1 to sensitive counterparts, leading to the transfer of chemoresistance phenotype. Importantly, the circulating level of exosomal UCH‐L1 before chemotherapy is adversely associated with the prognosis of breast cancer patients.^[^ ^76^ ^]^ In addition, the expression of the exosomal CD44 in DOX‐resistant breast cancer cells is higher than parental cells, suggesting that breast cancer cells may spread drug resistance capacity by the intercellular transfer of CD44 via exosomes.^[^ ^77^ ^]^ Phosphoribosyl pyrophosphate synthase 2 (PRPS2) in EVs enhances the resistance of nonsmall cell lung cancer (NSCLC) cells to cisplatin by promoting M2 polarization of macrophages.^[^ ^78^ ^]^ These studies indicate that proteins in EVs from cancer cells play important roles in spreading anticancer drug resistance.

### 4.3 EV DNA

Sansone et al. demonstrate that cancer cell‐derived exosomes transport mitochondrial DNA (mtDNA) and result in therapy resistance. They demonstrate that full mitochondrial genome is present in EVs from the blood of metastatic breast cancer patients who are resistant to hormonal therapy. EV mtDNA mediate therapy‐induced cancer stem‐like cells to recover from a dormant state to an activated one, leading to hormonal therapy resistance in an oxidative phosphorylation‐dependent manner.^[^ ^79^ ^]^

### 4.4 EV mRNAs

EVs derived mRNAs have been described as active regulators of cancer progression. ΔNp73, a TP73 gene isoform, is found to participate in the development of cancer in different stages.^[^ ^80^ ^]^ Soldevilla et al. demonstrate that the levels of ΔNp73 mRNA are higher in CRC‐derived exosomes than their parental cells and that exosomal ΔNp73 mRNA promotes oncogenic potential and chemoresistance in the recipient cells.^[^ ^81^ ^]^ Zhou et al. show that the mRNA levels of glycerol kinase 5 (GK5) are higher in gefitinib‐resistant (GR) cells than GR‐sensitive ones. Exosomal GK5 mRNA mediates gefitinib resistance while silencing of GK5 recovers drug sensitivity.^[^ ^82^ ^]^ EV‐delivered anaplastic lymphoma kinase (ALK) transcript has been proposed as a new mechanism for acquired drug resistance. Exosomal ALK promotes the transfer of drug resistance in melanoma cells by activating MAPK signaling pathway. Inhibition of ALK resensitizes the resistant cells to BRAF inhibitors.^[^ ^83^ ^]^ Lobb et al. suggest that exosomes from chemoresistant NSCLC cells transfer resistance to chemosensitive cells via ZEB1 mRNA (the major EMT transcription factor), suggesting a new mechanism for induction of chemoresistance.^[^ ^84^ ^]^

Wang et al. demonstrate that acute myeloid leukemia (AML) cell‐derived exosomes contain vascular endothelial growth factor (VEGF) and VEGF receptor (VEGFR) mRNA and could induce VEGFR expression in human umbilical vein endothelial cells (HUVECs). These exosomes promote the proangiogenic activity of HUVECs by enhancing glycolysis, resulting in vascular remodeling and acquisition of chemoresistance.^[^ ^85^ ^]^ Yu et al. demonstrate that glioma cells stimulate normal human astrocyte (NHA) into reactive astrocyte (RAS) in a noncontact manner and the amount of O^6^‐alkylguanine DNA alkyltransferase (MGMT) mRNA in exosomes released by RAS is significantly higher than that from nonreactive NHA. Importantly, MGMT‐negative glioma cells uptake exosomes from RAS and gain TMZ resistance by translating exogenous exosomal MGMT mRNA.^[^ ^86^ ^]^

PSMA3 and PSMA3‐AS1 are protein‐coding/noncoding antisense transcripts that are simultaneously disordered and positively correlated in MM cells. Further analysis suggests that PSMA3 and PSMA3‐AS1 are present in MM exosomes and play a unique role in delivering resistance to proteasome inhibitors (PI).^[^ ^87^ ^]^ Additionally, icotinib‐resistant NSCLC cells produce exosomes that contain MET mRNA and transfer it to surrounding icotinib‐sensitive cells. Interestingly, the detection of 10 mRNAs in plasma exosomes of NSCLC patients could predict icotinib therapy response.^[^ ^88^ ^]^ Xiao et al. suggest that FTO, an N^6^‐methyladenosine RNA demethylase, is remarkably increased in serum exosomes of patients with gefitinib resistance. Exosomal FTO upregulates ABCC10 expression in the recipient cells in a m^6^A‐dependent manner to promote gefitinib resistance, indicating that exosome‐mediated RNA modification represents another mechanism that promotes gefitinib resistance in NSCLC.^[^ ^89^ ^]^ DNMT1 (DNA methyltransferase 1) transcripts are enriched in exosomes from ovarian cancer cells, and coincubation with exosomes stimulates DNMT1 protein expression and renders the resistance of host cells to cisplatin. Furthermore, the drug‐resistant cells almost completely restore their sensitivity when cotreated with exosome inhibitor GW4869, indicating that exosome inhibitor can be combined with cisplatin to reverse drug resistance.^[^ ^90^ ^]^ Altogether, these studies suggest that mRNAs in EVs could be transferred into the recipient cells where they are translated into proteins to mediate the acquisition of chemoresistance.

### 4.5 EV Noncoding RNAs

EVs contain a variety of noncoding RNAs including miRNAs, long noncoding RNAs and circular RNAs. Mounting evidence indicates that EV‐mediated transfer of noncoding RNAs has great impact on the acquisition of drug resistance in cancer.^[^ ^91^ ^]^

#### 4.5.1 EV miRNAs

Previous studies suggest that miRNAs derived from EVs participate in the development of chemoresistance. It has been reported that miRNA‐containing EVs promote neuroblastoma (NBL) chemoresistance. Specifically, NBL triggers the educational process of human monocytes through exosomal miR‐21, leading to a toll‐like receptor 8 (TLR8) and NF‐кB‐dependent upregulation of miR‐155 in monocytes. Then, monocytes derived exosomal miR‐155 target telomeric repeat binding factor 1 (TERF1), a component of the shelterin complex, in NBL cells and induce the acquisition of cisplatin resistance.^[^ ^92^ ^]^ Additionally, exosome‐transmitted miR‐155 facilitates the cisplatin resistance in OSCC cells.^[^ ^93^ ^]^ Shen et al. demonstrate that breast cancer cells could release various EV miRNAs after chemotherapy, such as miR‐203a‐3p, miR‐195‐5p, and miR‐9‐5p, which concurrently targets the transcription factor One Cut Homeobox 2 (ONECUT2), inducing cancer stem cell‐like features and chemotherapy resistance.^[^ ^94^ ^]^

Increasing evidence suggests that miRNAs are enriched in EVs and have diverse roles in radiotherapy resistance. For instance, a recent study has investigated the miRNA profiling of matched pre‐ and postradiation treatment GBM samples from the same patient, and has identified the mostly altered miRNA, miR‐603. Ionizing radiation (IR) induces cellular export of miR‐603 through EV release, thereby decreasing IGF1 and IGF1R expression, which in turn promotes cancer stem‐cell state and acquires radiation resistance in GBM.^[^ ^95^ ^]^ In addition, the researchers have identified differentially expressed miRNAs in serum of lung cancer patients before and after radiotherapy and shown that miR‐208a is the only upregulated miRNA in the serum after radiotherapy. MiR‐208a can be transported by exosomes and forced expression of miR‐208a induces radioresistance via targeting p21 and activating the Akt/mTOR pathway in lung cancer cells.^[^ ^96^ ^]^ Similarly, Chen et al. demonstrate that hypoxic cancer cell‐derived exosomal miR‐340‐5p confers radioresistance in esophageal squamous cell carcinoma (ESCC) by targeting KLF10/UVRAG axis. The high levels of circulating exosomal miR‐340‐5p in ESCC patients are associated with a poorer radiotherapy response and prognosis.^[^ ^97^ ^]^ Moreover, Jiang et al. demonstrate that exosomal miR‐194‐5p from radiation‐induced dying cells enhances the DNA damage response and potentiates the survival of cancer repopulating cells by targeting transcription factor E2F3, leading to radiotherapy failure.^[^ ^98^ ^]^

MiRNA profiling analysis reveals that miR‐30b‐3p is upregulated in hypoxic glioblastoma stem cells derived EVs, due to HIF1*α* and STAT3‐meidated transcriptional activation, which induces TMZ resistance in the recipient cells by downregulating the expression of tumor suppressor gene RHOB (ras homolog family member B).^[^ ^99^ ^]^ A recent study suggests that pancreatic cancer stem cells transfer the drug‐resistant trait to GEM‐sensitive cancer cells by transmitting miR‐210 via exosomes.^[^ ^100^ ^]^ Exosomes from cisplatin‐resistant gastric cancer cells enhance stemness properties and resistance of the corresponding parental cells via delivering miR‐500a‐3p and targeting FBXW7.^[^ ^101^ ^]^ Patel et al. suggest that the conditioned media of GEM‐treated pancreatic cancer cells, specifically its EV components, confer chemoresistance to pancreatic cancer cells. Reactive oxygen species (ROS)‐detoxifying enzymes catalase (CAT) and superoxide dismutase 2 increase in pancreatic cancer cells after GEM‐Exo treatment, leading to acquired drug resistance, possibly by transferring their transcripts and miR‐155‐mediated downregulation of deoxycytidine kinase, a GEM‐metabolizing enzyme.^[^ ^102^ ^]^ EVs from sorafenib‐resistant renal cell carcinoma (RCC) cells shuttle miR‐31‐5p to sensitive cells by directly targeting MutL homolog1 (MLH1), thereby spreading drug resistance.^[^ ^103^ ^]^ Exosomes transfer miR‐222‐3p from GEM‐resistant NSCLC cells to the recipient cells, in which exosomal miR‐222‐3p targets SOCS3 to enhance GEM resistance.^[^ ^104^ ^]^ EV‐delivered miR‐761 enhances pazopanib resistance in synovial sarcoma. Specifically, miR‐761 targets thyroid hormone receptor interactor 6 (TRIP6), lamin A/C (LMNA), and NAD‐dependent protein deacetylase sirtuin‐3 (SIRT3) to increase resistance to chemotherapeutic agents.^[^ ^105^ ^]^ Moreover, exosomal miR‐46146 mediates oxaliplatin resistance of CRCs by targeting PDCD10.^[^ ^106^ ^]^ Exosomal miR‐21 promotes 5‐FU resistance of CRCs by targeting PDCD4 and transfers cisplatin resistance of OSCC by targeting PTEN and PDCD4.^[^ ^107^ ^]^

Exosomes shed by EGFR T790M‐mutant NSCLC cells transfer gefitinib resistance to sensitive cells through the activation of PI3K/Akt pathways. RNA sequencing confirms that miR‐3648 and miR‐522‐3p are the two most differentially expressed miRNAs in exosomes that induce gefitinib resistance.^[^ ^108^ ^]^ Exosomes from cisplatin‐resistant NSCLC cells contain miR‐425‐3p, which facilitates autophagy in the sensitive cells by targeting AKT1, eventually leading to chemoresistance.^[^ ^109^ ^]^ Similarly, exosomal miR‐423‐5p enhances cisplatin resistance in triple‐negative breast cancer.^[^ ^110^ ^]^ NSCLC cells transfer miR‐214 and miR‐100‐5p to sensitive cells via exosomes to mediate resistance to gefitinib and cisplatin, respectively.^[^ ^111^ ^]^ Kwok et al. demonstrate that EVs from ALK‐TKI‐resistant subclones induce drug resistance in the sensitive cells via transferring miR‐21‐5p and miR‐486‐3p.^[^ ^112^ ^]^ Jing et al. suggest that miR‐769‐5p is highly expressed in serum exosomes of cisplatin‐resistant patients and exosomal miR‐769‐5p targets CASP9 to suppress caspase pathway activation and p53 stability.^[^ ^113^ ^]^ Exosomal miR‐501 confers resistance to DOX in sensitive gastric cancer cells via inhibition of apoptosis and exosomal miR‐155‐5p from PTX‐resistant gastric cancer cells induce chemoresistance in the sensitive cells by targeting GATA3 and TP53INP1.^[^ ^114^ ^]^ The levels of exosomal miR‐1238 are higher in TMZ‐resistant GBM cells than sensitive cells and exosomal miR‐1238 induces TMZ resistance by targeting caveolin‐1.^[^ ^115^ ^]^ Exosomal miR‐32‐5p suppresses PTEN and activates PI3K/Akt pathway to induce multidrug resistance in hepatocellular carcinoma (HCC). Furthermore, high levels of exosomal miR‐32‐5p and low levels of PTEN are positively associated with unsatisfactory prognosis.^[^ ^116^ ^]^ EVs derived miRNAs also promote multidrug resistance in AML cell, in which two miRNAs, miR‐19b and miR‐20a, are differentially expressed between chemosensitive and chemoresistant counterparts.^[^ ^117^ ^]^

#### 4.5.2 EV lncRNAs

Long noncoding RNAs (lncRNAs) play diverse roles in cancer progression including therapy resistance. Previous studies suggest that lncRNAs are enriched in EVs and involved in the regulation of therapy response. Wang et al. demonstrate that HOTAIR is present in serum EVs of GBM patients and EVs derived HOTAIR confers TMZ resistance by regulating miR‐526b‐3p/EVA1 axis.^[^ ^118^ ^]^ In breast cancer, EVs transport lncRNA NEAT1 to upregulate KLF12 expression by sponging miR‐141‐3p, thereby promoting resistance to drugs such as cisplatin, PTX, and 5‐FU.^[^ ^119^ ^]^ Kwok et al. suggest that lncRNAs MEG3 and XIST are differentially expressed in EVs secreted by drug‐resistant ALK‐translocated lung cancer cells. These circulating EV‐RNAs can induce drug resistance in other subpopulations and maintain intratumoral heterogeneity.^[^ ^112^ ^]^

**Figure 3:**
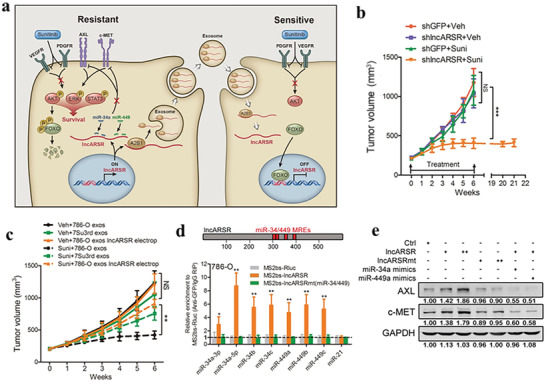
Exosome‐transmitted lncARSR promotes sunitinib resistance in renal cancer by acting as a competing endogenous RNA. a) Schematic diagram of lncARSR‐based signaling circuit in RCC sunitinib resistance. b) Tumor growth curves of 7Su3rd‐tumor‐bearing mice after different treatments. c) Tumor growth curves of 786‐O cells‐tumor‐bearing mice with intratumoral injection of indicated exosomes upon vehicle or sunitinib treatment. d) Upper: schematic outline of predicted binding sites for miR‐34 and miR‐449 on lncARSR. Lower: MS2‐based RIP assay with anti‐GFP antibody in 786‐O (upper) after different transfection. e) Western blot analysis of AXL and c‐MET in 786‐O cells transfected with different mimics and plasmids. Reproduced with permission.^[^ ^120^ ^]^ Copyright 2016, Elsevier.

The resistance to sunitinib is a main problem for advanced RCC therapy. Exosomal lncARSR promotes RCC resistance to sunitinib by competitively binding to miR‐34/miR‐449 and promoting the expression of AXL and c‐met in RCC cells (**Figure** **3**).^[^ ^120^ ^]^ Kang et al. demonstrate that lncRNA PART1 is highly expressed in gefitinib‐resistant ESCC cells and is transmitted to sensitive cells by exosomes, thus spreading gefitinib resistance. PART1 promotes resistance by competitively binding to miR‐129 to increase Bcl‐2 expression and knockdown of PART1 effectively improves gefitinib sensitivity in ESCC cells.^[^ ^121^ ^]^ Zhang et al. suggest that the EMT regulator ZEB1 transcriptionally activates oncogenic lncRNA SBF2‐AS1 expression and affects GBM resistance to TMZ. SBF2‐AS1 is enriched in GBM cells derived exosomes and acts as a ceRNA to regulate miR‐151a‐3p/XRCC4 (X‐ray repair cross complementing 4) axis, thus enhancing DNA damage repair and promoting drug resistance.^[^ ^122^ ^]^

LncRNA urothelial carcinoma‐associated 1 (UCA1) is upregulated in serum exosomes of cisplatin‐resistant ovarian cancer patients and UCA1 promotes cisplatin resistance through the regulation of miR‐143/FOSL2 pathway.^[^ ^123^ ^]^ In addition, exosome‐mediated transfer of UCA1 significantly increases tamoxifen resistance in estrogen receptor (ER) positive breast cancer cells.^[^ ^124^ ^]^ Wang et al. demonstrate that exosomal lncRNA HOTTIP contributes to cisplatin resistance by modulating HMGA1/miR‐218 axis in gastric cancer and enhances mitomycin resistance through the regulation of miR‐214/KPNA3 pathway in CRCs.^[^ ^125^ ^]^ Takahashi et al. suggest that lncRNA VLDLR is upregulated in HCC cells and exposure of HCC cells to different anticancer agents increases lnc‐VLDLR expression in their EVs. Incubation with these EVs increases the expression of lnc‐VLDLR in the recipient cells and inhibition of lnc‐VLDLR decreases cell viability and suppresses cell cycle progression.^[^ ^126^ ^]^ Chen et al. demonstrate that the upregulated linc‐VLDLR transmitted by EVs in esophageal cancer cells promotes drug resistance by upregulating ABCG2 expression.^[^ ^127^ ^]^ Treatment with TGF‐*β* or chemotherapeutic agents such as sorafenib upregulates lncRNA ROR expression in HCC cells and enriches them in EVs, which reduces chemotherapy‐induced cell death in the recipient cells.^[^ ^128^ ^]^ LncRNA AGAP2‐AS1 is transferred by exosomes to promote breast cancer resistance to trastuzumab, probably by regulating ATG10 expression and autophagy.^[^ ^129^ ^]^ Furthermore, lncRNA RP11 838N2.4 can be transmitted to sensitive cells via exosomes, thereby promoting erlotinib resistance in NSCLC.^[^ ^130^ ^]^ It has been reported that lncRNA H19 is higher in gefitinib‐resistant cells than sensitive cells and silencing of H19 promotes gefitinib‐induced cell cytotoxicity. H19 is also delivered by exosomes to promote gefitinib resistance in NSCLC cells.^[^ ^131^ ^]^ Pan and Zhou further suggest the role of exosomal H19 in erlotinib resistance in NSCLC, in which H19 promotes resistance via targeting miR‐615‐3p to upregulate ATG7 expression.^[^ ^132^ ^]^ Wang et al. show that exosome‐mediated transfer of H19 to sensitive cells leads to disseminate DOX resistance in breast cancer.^[^ ^133^ ^]^

Moreover, exosomes from NSCLC cells transfer lncRNA SOX2 overlapping transcript (SOX2‐OT) to macrophages to promote M2 polarization by targeting the miR‐627‐3p/Smads signaling pathway, which in turn enhances EGFR‐TKI resistance in NSCLC cells.^[^ ^134^ ^]^ TMZ‐associated lncRNA in GBM recurrence (lnc‐TALC) is enriched in GBM cells derived exosomes and transmitted to promote M2 polarization of the microglia. In turn, M2‐polarized microglia secret more amount of complement components C5/C5a, promoting the repair of DNA damage induced by TMZ and leading to chemotherapy resistance.^[^ ^135^ ^]^

#### 4.5.3 EV circRNAs

Increasing evidence indicates that exosomal circRNAs also regulate chemoresistance in cancer cells. Zhang et al. suggest that circUHRF1 is enriched in HCC cells derived exosomes and upregulates the expression of TIM‐3 via sponging miR‐449c‐5p, leading to resistance to anti‐PD1 immunotherapy.^[^ ^136^ ^]^ Hong et al. show that circ‐CPA4 regulates drug resistance in NSCLC by targeting let‐7/PD‐L1 axis. They demonstrate that NSCLC cells inactivate CD8^+^ T cells in a secreted PD‐L1‐dependent manner and circ‐CPA4 sponges let‐7 to upregulate the expression of exosomal PD‐L1. Circ‐CPA4 knockdown reactivates CD8^+^ T cells and promote NSCLC cell death.^[^ ^137^ ^]^ Chen et al. demonstrate that NSCLC cells derived circUSP7 sponges miR‐934 to upregulate SHP2 (src homology region 2‐containing protein tyrosine phosphatase 2) expression, thereby inhibiting CD8^+^ T cell function and promoting resistance to anti‐PD1 immunotherapy.^[^ ^138^ ^]^

Wang et al. demonstrate that exosomes from oxaliplatin‐resistant CRCs transfer circular RNA hsa_circ_0005963 (also termed as ciRS‐122) to sensitive cells to enhance glycolysis and drug resistance by regulating miR‐122/PKM2 axis.^[^ ^139^ ^]^ In addition, hsa_circ_0002130 is found to be upregulated in serum exosomes of osimertinib‐resistant NSCLC patients. It promotes osimertinib resistance by sponging miR‐498 to regulate the expression of glycolysis‐related genes.^[^ ^140^ ^]^ Hu et al. reveal that circ_UBE2D2 is upregulated in tamoxifen‐resistant breast cancer and circ_UBE2D2 can be transmitted by exosomes to sensitive cells, whereby it interacts with miR‐200a‐3p to enhance resistance to tamoxifen.^[^ ^141^ ^]^ Microarray analysis has identified 105 upregulated and 34 downregulated circRNAs in exosomes of FOLFOX‐resistant CRCs, in which the upregulation of exosomal hsa_circ_0000338 could predict chemoresistance in CRCs and knockdown of circ_0000338 restores CRC chemosensitivity.^[^ ^91c^ ^]^ In addition, circRNA‐SORE is overexpressed in sorafenib‐resistant HCC cells and exosomal circRNA‐SORE spreads sorafenib resistance among HCC cells via the stabilization of YBX1, a master gene regulator.^[^ ^142^ ^]^

Circ_0000337 in exosomes from cisplatin‐resistant esophageal cancer cells have been confirmed to accelerate drug resistance of sensitive cells partly by regulating the miR‐377‐3p/JAK2 axis.^[^ ^143^ ^]^ The levels of hsa_circ_0014235 are notably elevated in serum exosomes of patients with NSCLC. Exosome‐transmitted hsa_circ_0014235 promotes NSCLC cell resistance to cisplatin by regulating the miR‐520a‐5p/CDK4 axis.^[^ ^144^ ^]^ Luo and Gui suggest that circFoxp1 is an oncogene that confers ovarian cancer resistance to cisplatin by targeting miR‐22 and miR150‐3p to upregulate the expression of CEBPG (CCAAT enhancer binding protein gamma) and FMNL3 (formin like 3).^[^ ^145^ ^]^ The upregulation of circFoxp1 in serum exosomes from ovarian cancer patients may serve as an independent factor for predicting adverse survival outcome and drug resistance. Zhao et al. suggest that Cdr1as is decreased in serum exosomes of ovarian cancer patients and downregulation of Cdr1as promotes miR‐1270/SCAI (suppressor of cancer cell invasion) axis, leading to cisplatin resistance.^[^ ^146^ ^]^ Hypoxia profoundly contributes to chemoresistance of prostate cancer. A recent study indicates that exosomal circZNF91 mediates the signal transmission between hypoxic and normoxic prostate cancer cells to transmit chemoresistance. Mechanistically, hypoxia upregulates circZNF91 expression and circZNF91 is transported to normoxic prostate cancer cells via exosomes to increase the expression of deacetylase sirtuin1 (SIRT1) by sponging miR‐23b‐3p.^[^ ^147^ ^]^ Collectively, these studies indicate that EV‐circRNAs serve as a key mediator of chemoresistance in various cancers.

## 5 TME Cells Derived EVs in the Regulation of Drug Resistance

Cancer development and progression relies on the communication between cancer cells and noncancer cells in TME, which is a complex and dynamic system that consists of distinct noncancer cells such as stromal cells, immune cells, and endothelial cells.^[^ ^21^ ^]^ Previous studies have confirmed the multiple layers of interactions between cancer cells and TME cells, which contributes to many malignant behaviors of cancer cells.^[^ ^38^, ^148^ ^]^ By transporting biological active substances (proteins, nucleic acids, lipids, etc.), EVs derived from TME cells also have an important impact on the acquisition of drug resistance in cancer cells.^[^ ^120^, ^149^ ^]^

### 5.1 Mesenchymal Stem Cells (MSCs)

MSCs are a group of nonhematopoietic pluripotent stem cells with high self‐renewal and multidifferentiation abilities.^[^ ^150^ ^]^ Increasing evidence suggests that MSCs derived EVs promote therapy resistance through mediating intracellular communication.^[^ ^151^ ^]^ For example, in dormant breast cancer (BRCA), BRCA cells prime MSCs to release EVs containing miR‐222/223, which promotes quiescence in a subset of BRCA cells and confers drug resistance.^[^ ^152^ ^]^ Similarly, Exosomal miR‐23b from bone marrow MSCs (BMSCs) is shown to induce breast cancer resistance to docetaxel.^[^ ^153^ ^]^ In addition, EVs from BMSCs mediate drug resistance in MM cells.^[^ ^154^ ^]^ Exosomes from BMSCs carry miR‐155 to promote stemness in MM cells and upregulate drug resistance genes such as MRP1, ABCG2, and P‐gp, finally increasing drug resistance.^[^ ^155^ ^]^ When received repeated therapy of cytarabine and AC220 (an FLT3 inhibitor), MSCs derived exosomes from AML patients show high levels of miR‐155 and miR‐375, which confers drug resistance.^[^ ^156^ ^]^ MSCs derived EVs have also been reported as important mediators of gastric cancer drug resistance. The study from our group suggests that MSCs derived EVs could be uptaken by gastric cancer cells to activate CaMKs (calcium/calmodulin‐dependent protein kinases) and Raf/MEK/ERK kinase cascades, which enhances the expression of multidrug resistance‐related genes in gastric cancer cells and eventually leads to chemotherapy resistance.^[^ ^157^ ^]^ EVs from BMSCs transfer resistance of MM cells to protease inhibitor bortezomib through the PSMA3‐AS1/PSMA3 signaling, wherein PSMA3‐AS1 forms a duplex with pre‐PSMA3 mRNA to enhance its stability and translation.^[^ ^87^ ^]^ Moreover, EVs from BMSCs decrease the sensitivity of chronic myeloid leukemia (CML) cells to tyrosine kinase inhibitors, resulting in drug resistance.^[^ ^158^ ^]^ EVs from BMSCs also increase the therapy resistance of chronic lymphocytic leukemia cells to several drugs such as fludarabine, ibrutinib, idelalisib, and venetoclax.^[^ ^159^ ^]^ These studies suggest that MSCs derived EVs are an important mediator of TME‐induced drug resistance.

### 5.2 Stromal Cells

Wang et al. demonstrate that bone marrow stromal cells induce MM cells to develop resistance to bortezomib through secretion of exosomes. Stromal cells derived exosomes induce bortezomib resistance by activating survival‐related signaling pathways, including JNK, p38, and Akt pathways.^[^ ^154^ ^]^ In addition, loss of SIRT1 causes senescent phenotype in stromal cells and promotes their synthesis and secretion of EVs.^[^ ^160^ ^]^ The senescent stromal cells derived EVs, when uptaken by the recipient cells, increase the expression of ABCB4 to confer drug resistance.^[^ ^161^ ^]^ Boelens et al. suggest that stromal cells transfer exosomes to breast cancer cells, which activates STAT1/NOTCH3 signaling via pattern recognition receptor RIG‐I and expands tumor‐initiating cells, ultimately driving resistance to chemotherapy and radiotherapy.^[^ ^162^ ^]^

### 5.3 Cancer‐Associated Fibroblasts (CAFs)

EVs participate in the dynamic interaction between CAFs and cancer cells by transmitting paracrine signals. The transfer of miRNA by EVs from CAFs to cancer cells is involved in chemotherapy resistance. Previous studies suggest that ferroptosis is related to chemotherapy resistance. In gastric cancer, cisplatin and PTX induce CAFs to secret miR‐522 by activating the USP7 (ubiquitin‐specific protease 7)/hnRNPA1 (heterogeneous nuclear ribonucleoprotein A1) axis. Exosomal miR‐522 from CAFs inhibits the expression of arachidonate lipoxygenase 15 (ALOX15), an inhibitor of ferroptosis, in gastric cancer cells, leading to reduced accumulation of lipid‐ROS in cancer cells and ultimately a decreased sensitivity to chemotherapy.^[^ ^163^ ^]^ CAFs transfer functional miR‐196a to head and neck cancer cells through exosomes and exosomal miR‐196a targets ING5 and CDKN1B to confer cisplatin resistance. The removal of exosomes or knockdown of miR‐196a in CAFs functionally restores cisplatin sensitivity.^[^ ^164^ ^]^ Richards et al. show that CAFs release exosomes upon exposure to GEM, which increases Snail expression in PDAC cells and induce GEM resistance.^[^ ^165^ ^]^ In addition, miRNA‐106b from CAFs is transferred to PDAC cells through EVs, which causes cancer cells to be resistant to GEM by targeting TP53INP1.^[^ ^166^ ^]^ Moreover, miR‐21 has been reported to be involved in CAF EV‐induced GEM resistance in pancreatic cancer.^[^ ^167^ ^]^ In colorectal cancer, exosomes from CAFs transfer miR‐92a‐3p to CRCs to activate Wnt/*β*‐catenin pathway by inhibiting FBXW7 and MOAP1, contributing to 5‐FU/L‐OHP resistance in CRCs.^[^ ^168^ ^]^ Chen et al. demonstrate that exosome‐mediated transfer of miR‐93‐5p from CAFs to CRCs confers radioresistance by downregulating FOXA1 (forkhead box A1) and upregulating TGFB3.^[^ ^169^ ^]^ Shi et al. suggest that miR‐20a is enriched in CAFs derived exosomes and exosomal miR‐20a targets PTEN/PI3K/Akt pathway to promote cisplatin resistance of NSCLC.^[^ ^170^ ^]^

In addition to miRNAs, lncRNAs also serve as important players in CAF EV‐mediated drug resistance. H19 is enriched in CAFs derived exosomes and it promotes the stemness and chemoresistance of colorectal cancer stem cells by acting as a competing endogenous RNA for miR‐141.^[^ ^171^ ^]^ Deng et al. demonstrate that exosomes from CAFs transfer lncRNA CCAL to CRCs to activate Wnt/*β*‐catenin signaling pathway through HuR, leading to resistance to oxaliplatin and 5‐FU.^[^ ^172^ ^]^ LINC00355 in exosomes from CAFs promotes cisplatin resistance of bladder cancer cells by regulating the miR‐34b‐5p/ABCB1 axis.^[^ ^173^ ^]^ Exosomes from ESCC cells transfer lncRNA POU3F3 to normal fibroblasts to induce their conversion to CAFs, which in turn promotes cisplatin resistance of ESCC cells by secreting interleukin 6 (IL‐6).^[^ ^174^ ^]^

**Figure 4:**
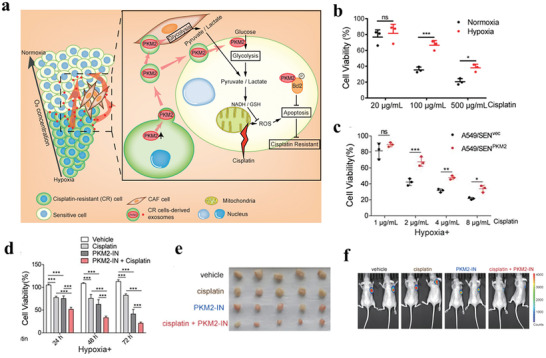
Hypoxia‐induced exosomes transmit cisplatin resistance to sensitive NSCLC cells by delivering PKM2. a) Schematic illustration of hypoxia‐exosomal PKM2 in promoting cisplatin‐resistance of NSCLC cells. b) Cell viability of A549/CR cells treated in normoxic or hypoxic conditions and treated with different concentrations of cisplatin. c) Cell viability of A549/SEN^vec^ and A549/SEN^PKM2^ cells. d) Cell viability of A549/CR cells with different treatments. e) Tumor tissues obtained from tumor‐bearing mice after different treatments. f) In vivo bioluminescence imaging for this model. Reproduced with permission.^[^ ^176^ ^]^ Copyright 2021, Ivyspring International Publisher.

In addition, EVs derived from CAFs mediate drug resistance through the transfer of proteins and circRNAs. Fibroblasts can reprogram differentiated cells to CSCs by transferring Wnts via exosomes, thus promoting chemoresistance in CRCs.^[^ ^175^ ^]^ Hypoxia induces cisplatin resistance in NSCLC cells via exosomal PKM2. Hypoxia‐induced exosomal PKM2 not only promotes glycolysis in NSCLC cells to produce reductive metabolites, thus neutralizing cisplatin‐induced ROS, but also reprograms CAFs to create an acidic microenvironment that promotes cisplatin resistance^[^ ^176^ ^]^ (**Figure** **4**). Uchihara et al. suggest that CAFs derived EVs contain Annexin A6 and enhance gastric cancer drug resistance by activating *β*1 integrin‐adhesion kinase (FAK)‐YAP signaling.^[^ ^177^ ^]^ Recently, Qu et al. demonstrate that CAFs derived cricN4BP2L2 binds to EIF4A3 to activate PI3K/Akt/mTOR pathway, inducing oxaliplatin resistance in CRCs.^[^ ^178^ ^]^ These findings suggest that CAFs in TME play an active role in regulating therapy resistance through EVs.

### 5.4 Macrophages

Macrophages are the most abundant noncancer cell population in the TME. Macrophages are divided into two subtypes: M1 macrophages with antitumor functions and M2 macrophages with protumor functions.^[^ ^179^ ^]^ The resident macrophages in cancer stroma are termed as tumor‐associated macrophages (TAMs), usually presenting M2 phenotype and playing key roles in regulating cancer drug resistance.^[^ ^180^ ^]^ EVs derived from TAMs mediate cancer drug resistance through a variety of bioactive molecules, of which miRNA plays a dominant role. For example, TAMs transfer miR‐27a‐3p, miR‐22‐3p, and miR‐221‐3p into glioblastoma stem cells through exosomes, inducing radiotherapy resistance by targeting CHD7 and regulating RelB/P50 and STAT3 pathways to trigger proneural‐to‐mesenchymal transition.^[^ ^181^ ^]^ Exosomal miR‐365 from TAMs also contributes to GEM resistance in PDAC by upregulating the pool of triphospho‐nucleotide and inducing the expression of enzyme cytidine deaminase.^[^ ^182^ ^]^ Moreover, TAM exosomes derived miR‐223 and miR‐21 induce cisplatin resistance in gastric cancer and ovarian cancer, respectively.^[^ ^183^ ^]^

LncRNAs in macrophage EVs also mediate drug resistance in cancer cells. LncRNA CRNDE is enriched in exosomes of M2 macrophages and transferred from M2 macrophages to gastric cancer cells via exosomes. The high level of lncRNA CRNDE in gastric cancer cells reduces their sensitivity to cisplatin by inhibiting PTEN expression.^[^ ^184^ ^]^ In addition, TAMs transmit HIF‐1*α*‐stabilizing long noncoding RNA (HISLA) to breast cancer cells by EVs to enhance their aerobic glycolysis and drug resistance. Specifically, HISLA inhibits the hydroxylation and degradation of HIF‐1*α* by blocking the interaction of HIF‐1*α* with PHD2.^[^ ^185^ ^]^

In addition, CHI3L1 (chitinase 3‐like‐1) and FN1 (fibronectin 1) are identified as the two most enriched proteins in macrophage‐derived EVs, which induces GEM resistance by activating ERK pathway in PDAC cells.^[^ ^186^ ^]^ There are also reports showing that TAMs secrete a large amount of transcription factor GATA3 through EVs, which regulates the polarization of macrophages and promote the resistance of ovarian cancer cells to cisplatin.^[^ ^187^ ^]^ Overall, EVs derived from macrophages induce therapeutic resistance in various cancers through multiple mechanisms.

### 5.5 Myeloid‐Derived Suppressor Cells (MDSCs) and Treg

MDSCs are a group of heterogeneous myeloid cells with immunosuppressive functions. The high number of MDSCs represents a possible mechanism of resistance to immunotherapy in cancer patients. Huber et al. demonstrate that melanoma EVs induce the conversion of monocytes into monocytic MDSC through miRNA‐mediated transcriptional regulation (namely, miR‐146a, miR‐100, miR‐125b, and miR‐155). Moreover, these miRNAs are enriched in the plasma of melanoma patients and are closely related to the clinical efficacy of immune checkpoint inhibitors (ICIs).^[^ ^188^ ^]^ Recent studies also suggest that MDSCs derived EVs regulate the efficacy of chemotherapy.^[^ ^189^ ^]^ Exosomal miR‐126a released from DOX‐induced MDSCs confers the resistance of breast cancer cells to DOX therapy and promotes lung metastasis.^[^ ^190^ ^]^

Regulatory T cells (Treg) are known for their immunosuppressive effects, and the relationship between Treg EVs and chemotherapy resistance has not been well characterized. A recent study shows that exosomal miR‐208b secreted by colon cancer cells is transferred to the recipient T cells whereby it targets programmed cell death factor 4 (PDCD4) to promote Treg expansion, which in turn induces oxaliplatin resistance and cancer growth.^[^ ^191^ ^]^

### 5.6 Endothelial Cells, Adipocytes, and Hepatic Stellate Cells

In addition to the cells mentioned above, blood vessel endothelial cells are another type of TME cells that can also lead to drug resistance.^[^ ^192^ ^]^ Although antiangiogenesis therapy has shown effects against multiple malignancies, their efficacy is limited by subsequent cancer vasculogenesis and progression. The vasculogenesis inhibitor vandetanib is found to induce the release of VEGF‐enriching exosomes, which significantly promotes endothelial vessel formation and angiogenic mimicry in mouse HCC, indicating that the vasculogenesis and progression after AATs are due to the crosstalk between endothelial cells and cancer cells through VEGF‐rich exosomes.^[^ ^193^ ^]^ In addition, EVs also contain a variety of membrane‐associated proteins with angiogenic properties.^[^ ^194^ ^]^ Sato et al. demonstrate that EPHB2 (ephrin type B receptor 2) is present on small EVs secreted by head and neck cancer cells and it stimulates cancer angiogenesis by interacting with ephrin‐B2 on the surface of endothelial cells to activate the STAT3 signaling pathway, which exacerbates the resistance to antiangiogenesis therapy.^[^ ^195^ ^]^ In turn, it has also been reported that human microvascular endothelial cells promote chemotherapy resistance in NPC cells through the secretion of EVs.^[^ ^196^ ^]^

Adipocytes have been regarded as an important player in cancer progression. Liu et al. demonstrate that exosomes from adipocytes transfer miR‐23a/b to HCC cells to confer 5‐FU resistance by targeting the VHL/HIF axis.^[^ ^197^ ^]^ MiR‐21 is enriched in cancer‐associated adipocytes (CAAs) derived exosomes and is transferred from CAAs to ovarian cancer cells, promoting chemotherapy resistance by regulating APAF1.^[^ ^198^ ^]^ Wang et al. suggest that MM cells could promote the sorting of LOC606724 and SNHG1 into adipocyte exosomes through METTL7A‐mediated LncRNA m^6^A methylation. In turn, exosomal LOC606724 and SNHG1 from adipocytes prevent MM cells from chemotherapeutic drug‐induced apoptotic damage, leading to therapy resistance.^[^ ^199^ ^]^ Microsomal triglyceride transfer protein (MTTP), an inhibitor of ferroptosis, is enriched in plasma exosomes from colorectal cancer patients. Adipocyte‐derived exosomal MTTP inhibits ferroptosis and promotes chemoresistance to oxaliplatin in colorectal cancer through the MTTP/PRAP1/ZEB1 axis.^[^ ^200^ ^]^

The activated hepatic stellate cells (aHSCs) in TME have been identified to promote cancer development.^[^ ^201^ ^]^ Exosomes derived from CRCs induce HSCs to secrete excess IL‐6, which activates the IL‐6/STAT3 pathway to enhance lactate metabolism in CRCs and upregulates the expression of monocarboxylate transporters 1 (MCT1) and lactate dehydrogenase B (LDHB), thereby conferring resistance to irinotecan.^[^ ^202^ ^]^

### 5.7 Hypoxia

Hypoxia is common in cancer and is closely associated with drug resistance. EVs released in the hypoxic tumor microenvironment transmit signals between cancer cells and noncancer cells.^[^ ^203^ ^]^ Under hypoxic conditions, ovarian cancer cells release more exosomes by upregulating Rab27a and represent a more secretory lysosomal phenotype. The hypoxia‐induced exosomes contain more potent oncogenic proteins, such as STAT3 and FAS, inducing cell resistance to cisplatin.^[^ ^204^ ^]^ The recent evidence indicates that exosomal miR‐301a (exo‐miR‐301a) from hypoxic GBM cells could be transferred to the normoxic cells. Hypoxic exo‐miR‐301a targets TCEAL7 to activate Wnt/*β*‐catenin signaling, thus promoting radiation resistance.^[^ ^205^ ^]^ Exosomal miR‐21 derived from hypoxic NSCLC cells promotes the resistance of normoxic cells to cisplatin by downregulating PTEN.^[^ ^206^ ^]^ MiR‐223 is enriched in TAM exosomes in response to hypoxia and internalized by ovarian cancer cells to induce a chemoresistance phenotype through the PTEN‐PI3K/Akt pathway.^[^ ^183b^ ^]^ Therefore, hypoxia promotes the release of EVs with specific cargos to mediate drug resistance in cancer.

## 6 The Implications of EVs in Monitoring Cancer Therapy Response

EVs are considered to have the potential to monitor cancer progression. Based on the many unique bioactive cargoes contained in EVs, such as proteins, nucleic acids, etc., researchers have developed many novel cancer biomarkers, opening up new and interesting opportunities for cancer diagnosis and prognosis (**Table** **2**).

**Table 2:** EV cargos as potential biomarkers for predicting cancer therapy response

Cargo type	Molecules	Change	Detection method	Clinical significance	Refs.
DNA	DNMT1	Upregulation	Gene array	Predict increased cisplatin resistance	[90]
	EGFR	p.T790 M mutation	Peptide nucleic acid (PNA)‐mediated PCR clamping	Higher efficiency than tissue biopsy to determine acquired resistance patients	[229]
	MGMT	*MGMT* genomic rearrangements	RNA‐sequencing	Represent an early detection marker of tumor recurrence in patients treated with TMZ	[230]
RNA	CDK4, TK1, and CDK9 mRNA	Upregulation	Digital droplet PCR (ddPCR)	High baseline CDK4 levels are associated with longer PFS after therapy, TK1 and CDK9 levels are associated with clinical resistance	[213]
	GSTP1 mRNA	Upregulation	RT‐PCR	Predict increased resistance to chemotherapeutics	[214]
	miR‐21‐3p, miR‐21‐5p, and miR‐891‐5p	Upregulation	RT‐PCR	Correlate with risk of ovarian cancer relapse	[221]
	miR‐34a	Downregulation	Taqman miRNA low density arrays (TLDA)	Correlate with docetaxel response and cancer progression	[223]
	miR‐29c, miR‐342‐3p, and let‐7b	Downregulation	RT‐PCR	Differentiate poor responders from good responders	[224]
	miR‐181a, miR‐1908, miR‐21, miR‐486, and miR‐223	Upregulation	Next‐generation sequencing	Predict primary platinum‐resistance at the primary diagnosis of ovarian cancer	[227]
	CD44v8‐10 mRNA	Upregulation	RT‐digital PCR	Diagnostic marker for docetaxel‐resistant castration‐resistant prostate cancer	[231]
	MGMT and APNG mRNA	Upregulation	Immunomagnetic capture by a microfluidic chip and PCR	Predictor variables for treatment outcomes	[232]
Protein	TGF*β*1	Upregulation	ELISA	Correlate with patients' response versus resistance to HER2‐targeted drugs	[66]
	PD‐L1	Upregulation	HOLMES‐ExoPD‐L1	Predict immunotherapy response	[208]
	TRPC5	Upregulation	Flow cytometry	Correlate with tumor response to chemotherapy and acquired chemoresistance	[210]
	P‐glycoprotein (P‐gp) and phosphatidylserine (PS)	Upregulation	Flow cytometric analysis	Correlate with disease progression and treatment unresponsiveness	[212]
	Total exosome proteins, TEX/total exosome ratios, total CD3^+^, CD3^−^PD‐L1^+^, and CD3 ^+^CD15s^+^ (Treg‐derived) exosomes	Upregulation	Exclusion chromatography, immunocapture, and on‐bead flow cytometry	Evaluate therapy responses	[216]
	Melanoma chondroitin sulfate proteoglycan (MCSP), melanoma cell adhesion molecule (MCAM), low‐affinity nerve growth factor receptor (LNGFR), and receptor tyrosine protein kinase (ErbB3)	Upregulation	Nanomixing‐enhanced microchip and the multiplex surface‐enhanced Raman scattering (SERS) nanotag system	Infer the treatment response and obtain tumor cell‐specific information	[235]

### 6.1 EV Proteins

EVs derived proteins are critically involved in the regulation of therapy resistance and thus could be used as biomarkers for monitoring cancer therapy efficacy. Yu et al. show that EGFR expression in EGFR‐mutant NSCLC patient‐derived exosomes is more than tenfold higher than that in healthy controls and exosomal EGFR levels may reflect the alteration of cancer EGFR after therapy.^[^ ^207^ ^]^ Huang et al. have developed an efficient and sensitive quantitation method (HOLMES‐ExoPD‐L1) for exosomal PD‐L1 to predict cancer immunotherapy response. The circulating levels of exosomal PD‐L1 detected by this method can effectively distinguish cancer patients from healthy individuals, and are found to positively correlate with metastasis.^[^ ^208^ ^]^ Porcelli et al. demonstrate that the responders to ICIs have a significantly lower ratio of uPAR^+^ EVs than nonresponders. A high level of melanoma‐derived uPAR^+^ EVs is strongly associated with poor progression‐free and overall survival.^[^ ^209^ ^]^

The levels of TGF*β*1 in EVs are correlated with patients’ response to HER2‐targeted drugs in breast cancer.^[^ ^66^ ^]^ In addition, the level of circulating exosomal TRPC5 (cirExo‐TRPC5) is associated with that in breast cancer tissues and increased cirExo‐TRPC5 level after chemotherapy precedes progressive disease and strongly predicts acquired chemoresistance.^[^ ^210^ ^]^ Ciravolo et al. show that around 3/4 of advanced breast cancer patients have HER2^+^ EVs in the blood, which effectively sequesters trastuzumab monoclonal antibodies, thus impairing its therapeutic efficacy. The HER2^+^ EVs in the circulation of breast cancer patients may be used to predict trastuzumab therapy efficacy.^[^ ^211^ ^]^ Krishnan et al. suggest that circulating large EVs (specifically MPs) are useful for monitoring disease progression and MDR in myeloma, and the higher levels of P‐gp^+^ and phosphatidylserine (PS)^+^ MPs are correlated with disease progression and therapy failure. Therefore, circulating MPs could be used in liquid biopsy to monitor MDR and treatment failure in myeloma.^[^ ^212^ ^]^

The expression levels of thymidine kinase 1 (TK1) and cyclin‐dependent kinase 9 (CDK9) are upregulated in plasma‐derived exosomes and are positively correlated with resistance to CDK4/6 inhibitors in patients with metastatic breast cancer. Additionally, high levels of CDK4 mRNA are related to the response to palbociclib plus hormonal therapy, while the increase of TK1 and CDK9 mRNA copies is related to clinical resistance.^[^ ^213^ ^]^ Yang et al. suggest that the levels of glutathione S‐transferase P1 (GSTP1), a phase II metabolic enzyme, in serum exosomes of breast cancer patients who have received anthracycline/taxane‐based neoadjuvant chemotherapy are higher in the progressive/stable disease group than those in the partial/complete response group, suggesting the predictive role of GSTP1‐containing exosomes.^[^ ^214^ ^]^

Hypoxia is reported to cause changes in cell death/survival pathways, resulting in increased cell resistance to radiation. Thomas et al. demonstrate that the protein profiles of exosomes can characterize hypoxia‐induced radiation resistance in breast cancer.^[^ ^215^ ^]^ Theodoraki et al. have analyzed the predictive value of exosomal cargos for head and neck cancer recurrence during therapy. They suggest that in the recurred patients after ipilimumab therapy, the expression of total exosome proteins, cancer‐derived/total exosome ratios, the number of total CD3^+^, CD3^−^PD‐L1^+^, and CD3^+^CD15s^+^ (Treg‐derived) exosomes increases and that cancer‐derived and T cell‐derived circulating exosomes can be used to monitor patients’ responses to therapy.^[^ ^216^ ^]^ Stübiger et al. have analyzed the protein profiles of EVs from primary, metastatic, and drug‐resistant subclones and revealed that EV protein profiling may serve as a novel tool for minimally invasive monitoring of cancer chemotherapy.^[^ ^217^ ^]^ An et al. have compared the proteomes of exosomes from pancreatic cancer patients at different time points during treatment with those from healthy controls and have identified eight proteins that show global treatment‐specific changes, suggesting that the change of exosomal proteins during therapy could be an indicator for predicting therapy response.^[^ ^218^ ^]^

### 6.2 EV Nucleic Acids

Recent studies suggest that the circulating levels of EVs derived miRNAs can help to predict the efficacy of chemotherapy and evaluate the risk of drug resistance. Exosomal nucleic acids in the plasma and pleural fluid can potentially reflect the genomic changes in NSCLC patients who develop resistance to targeted EGFR inhibitor therapy. Kim et al. demonstrate that the combination of circulating and exosomal nucleic acid in the pleural fluid can help evaluate low‐abundant EGFR mutant copies in NSCLC.^[^ ^219^ ^]^ C‐Met activation increases miR‐130b levels and promotes cancer resistance to hormone ablation therapy. Evaluating the expression of Met and miR‐130b in circulating exosomes offers a new noninvasive tool for active surveillance and therapy monitoring of surgery candidates and advanced patients.^[^ ^220^ ^]^ MiR‐21‐3p, miR‐21‐5p, and miR‐891‐5p are enriched in EVs and contribute to carboplatin resistance in ovarian cancer. The detection of these miRNAs in EVs from biofluids could determine patient response to chemotherapy.^[^ ^221^ ^]^

Similarly, miR‐425‐3p is suggested as one of the most differentially expressed miRNAs in serum exosomes of platinum‐resistant patients compared to platinum‐sensitive ones. High levels of exosomal miR‐425‐3p are associated with low responsiveness and poor progression‐free survival in NSCLC patients, indicating that exosomal miR‐425‐3p may be a biomarker for predicating the response to platinum‐based chemotherapy.^[^ ^222^ ^]^ In prostate cancer, exosomal miR‐34a has been shown to promote cell resistance to docetaxel by regulating BCL‐2 and it could serve as a predictive biomarker for the therapeutic effect of docetaxel.^[^ ^223^ ^]^

The accumulation of MDSCs is a major obstacle to effective cancer immunotherapy. Huber et al. have identified several exosomal miRNAs (such as miR‐155, miR‐146a, miR‐125b, and miR‐100) that induce the conversion of monocytes into monocytic MDSCs and promote melanoma resistance to ICI treatment. The plasma levels of these exosomal miRNAs are tightly related to the clinical efficacy of ICIs, suggesting that MDSC‐related miRNAs may serve as a potential biomarker of a poor immunotherapy outcome.^[^ ^188^ ^]^ Morini et al. have compared the plasma exosomal miRNA profiles of high‐risk neuroblastoma patients before and after chemotherapy and identified a panel of three exosomal miRNAs that could distinguish good and poor responders.^[^ ^224^ ^]^ Zheng et al. have identified 114 EVs derived miRNAs to be deregulated in drug resistant head and neck cancer cells, including 44 downregulated miRNAs and 70 upregulated miRNAs.^[^ ^225^ ^]^ Exosomal miR‐99a‐5p and miR‐125b‐5p are upregulated in DLBCL and their increased levels in the serum of DLBCL patients are correlated with shorter progression‐free survival time, indicating its potential to predict chemotherapeutic efficacy.^[^ ^226^ ^]^

Kuhlmann et al. have analyzed EV‐related miRNAs in plasma from ovarian cancer patients who are resistant to platinum therapy and identified several differentially expressed miRNAs in plasma EVs such as miR‐181a, miR‐21, miR‐1908, miR‐486, and miR‐223. These EVs derived miRNAs have a high potential to be developed as predictors of platinum resistance.^[^ ^227^ ^]^ A recent study shows that trastuzumab‐resistant and sensitive patients could be distinguished via circulating exosomal miR‐155 and miR‐1246 as their upregulation is closely associated with shorter disease‐free survival in early‐stage patients and progression‐free survival in metastatic patients.^[^ ^228^ ^]^

In addition to miRNAs, other types of nucleic acids in EVs also have important implications in monitoring therapy efficacy and drug resistance. DNA in EVs from plasma and bronchoalveolar lavage fluid of NSCLC patients can be used for EGFR genotyping. EV DNA show tissue‐specific and ultrasensitive results compared to cell‐free DNA in acquired resistance patients, revealing that EV DNA may provide a more accurate, cheaper, and faster diagnostic result.^[^ ^229^ ^]^ Oldrini et al. suggest that MGMT genomic arrangement and TMZ resistance simultaneously occur in some recurrent glioma patients. Tumor‐derived exosomes contain MGMT gene fusions, which can be utilized as a predictor of tumor recurrence in the patients treated with TMZ.^[^ ^230^ ^]^

A recent study suggests that the levels of CD44v8‐10 protein and mRNA in exosomes are higher in docetaxel‐resistant prostate cancer cells than sensitive cells. The copy numbers of CD44v8‐10 mRNA in serum exosomes are higher in docetaxel‐resistant patients than docetaxel‐naïve patients and control males, suggesting that serum exosomal CD44v8‐10 mRNA could be a diagnostic biomarker for docetaxel resistance in prostate cancer patients.^[^ ^231^ ^]^ Shao et al. have developed a microfluidic chip for analyzing the mRNA levels of MGMT and APNG in circulating exosomes of GBM patients. They demonstrate that exosomal mRNA levels of these enzymes in the patients change considerably during treatment, proposing that chip‐based analysis of exosomal mRNA could predict drug resistance in GBM.^[^ ^232^ ^]^ Del Re et al. present a new method to detect androgen receptor splice variant 7 (AR‐V7) mRNA in EVs from the plasma of patients with castration‐resistant prostate cancer. They suggest that the levels of EVs‐AR‐V7 mRNA could predict hormone therapy resistance and its level is inversely associated with patients' overall survival.^[^ ^233^ ^]^

Shi et al. have performed transcriptomic profiling of plasma‐derived EVs from patients with metastatic melanoma receiving ICIs and revealed the presence of drivers for ICI resistance and melanoma progression. They suggest that EVs could serve as predictive biomarkers of ICI responsiveness, cancer persistence, and immune activation.^[^ ^234^ ^]^ The detection of EVs phenotype is also a promising tool for monitoring treatment response. By using a multiplex EV phenotyping chip, Wang et al. have monitored the phenotypic evolution of EVs in plasma of eight melanoma patients receiving targeted therapy and have revealed a specific EVs profile associated with the development of drug resistance.^[^ ^235^ ^]^

A variety of deregulated lncRNAs and circRNAs in EVs are involved in cancer drug resistance, and thus could be utilized as new biomarkers. Yang et al. show that circulating exosomal lncRNA UCA1 could predict the therapeutic efficacy of cetuximab in CRC patients. Compared with those with good response, the patients with poor response show considerably higher levels of circulating exosomal UCA1.^[^ ^236^ ^]^ A recent study shows that circ_0008928 confers cisplatin resistance in NSCLC by regulating miR‐488/HK2 axis and the levels of circ_0008928 are upregulated in serum exosomes of cisplatin‐resistant patients.^[^ ^237^ ^]^

## 7 The Potential of EVs in Overcoming Cancer Drug Resistance

**Figure 5:**
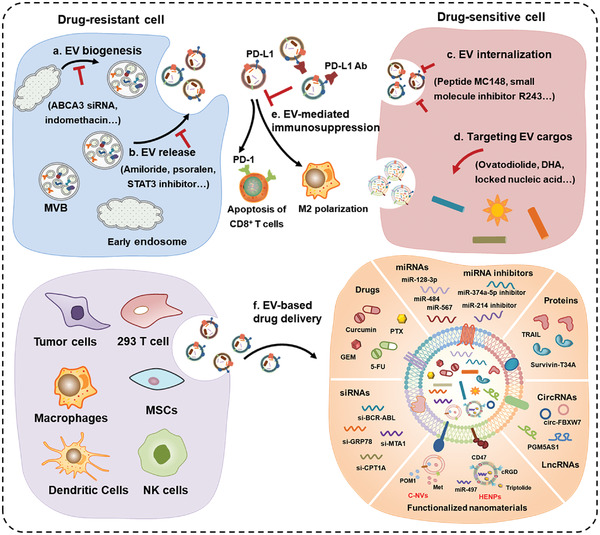
The strategies of utilizing EVs for overcoming cancer drug resistance. a) Inhibiting biogenesis of EVs. b) Inhibiting EVs secretion. c) Inhibiting internalization of EVs by target cells. d) Targeting EV cargos involved in cancer drug resistance (proteins, miRNA, and lncRNA). e) Targeting EVs to activate cancer immune response. f) As drug delivery tools to restore drug sensitivity (as carriers for anticancer proteins, siRNA, ncRNAs, drugs, and functionalized nanomaterials).

The critical role of EVs in conferring drug resistance implies that it may be used as a target for the reversal of drug resistance. Several relevant strategies such as altering the content of EVs and blocking EV‐mediated crosstalk in TME have been proposed and verified in preclinical studies. Moreover, the potential of EVs as a delivery tool for overcoming drug resistance has also been examined (**Figure** **5**).

### 7.1 EVs as Targets for the Reversal of Cancer Drug Resistance

Targeted inhibition of the signaling pathway regulated by EVs has been tested as a common strategy to overcome cancer drug resistance and to improve cancer therapy efficacy. In addition, EVs have been used as a vaccine adjuvant to trigger a strong anticancer response and thus to amplify the effect of cancer immunotherapy.

#### 7.1.1 Targeting EV‐Mediated Crosstalk in Cancer Drug Resistance

Up to now, three main strategies targeting drug resistance signaling pathways mediated by EVs have been developed to reverse cancer drug resistance: 1) inhibiting the biogenesis and release of EVs, 2) targeting EV cargos, and 3) inhibiting the internalization of EVs by target cells.^[^ ^21^ ^]^

Accumulating evidence suggests that EVs represent a new mechanism for cancer drug resistance as EVs transfer nucleic acids, proteins, drug transporters, and even drugs between cells.^[^ ^238^ ^]^ Therefore, interfering with the biogenesis and secretion of EVs is a promising method to overcome cancer drug resistance. For instance, ABCA3 promotes the secretion of EVs and the occurrence of drug resistance. The suppression of ABCA3 with siRNA or indomethacin inhibits the biogenesis of exosomes, which increases the intracellular retention of drugs, shifts the subcellular drug accumulation to prolong nuclear retention, and enhances the sensitivity of cancer cells to DOX and pixantrone.^[^ ^239^ ^]^ In addition to ABCA3, some potent oncoproteins, such as STAT3, also regulates EV biogenesis and secretion. Blocking the release of EVs with amiloride or STAT3 inhibitor, when combined with cisplatin treatment, leads to an increased apoptosis and decreased cell proliferation in chemoresistant ovarian cancer cells.^[^ ^204^ ^]^ In addition, psoralen, an active ingredient from Fructus Psoraleae with anticancer activity, reduces the biogenesis and release of exosomes by regulating PPAR and p53 signaling pathways and thus overcomes exosome‐mediated resistance to DOX in breast cancer.^[^ ^240^ ^]^ As previously described, senescent stromal cells contribute to cancer resistance by producing large amounts of EVs through the inhibition of SIRT1. Therefore, activating SIRT1 with SRT2104 prevents drug resistance by inhibiting the production of EVs in senescent stromal cells.^[^ ^161^ ^]^ Furthermore, PI3K/Akt signaling pathway regulates the subcellular localization of ABCG2. Inhibition of the Akt signaling pathway causes ABCG2 to gradually relocate from EV membrane to the cytoplasm. The intracellular retention of ABCG2 leads to a decreased EV amount, leading to the reversal of multidrug resistance in breast cancer cells.^[^ ^48^ ^]^ System Xc^−^ promotes exosome biogenesis and exosomal communication between BMSCs and MM cells to induce bortezomib resistance. The use of a system Xc^−^ inhibitor, sulfasalazine, could reduce MM resistance to bortezomib induced by BMSCs, thus enhancing anti‐MM effects.^[^ ^241^ ^]^

Targeting EV cargos that are involved in cancer drug resistance may be more practical than systematically blocking the release of EVs. Treatment with docosahexaenoic acid (DHA), a natural compound with anticancer activity, could alter the miRNA profile of exosomes from breast cancer cells. Exosomes from DHA‐treated cells transfer miR‐23b and miR‐320b to endothelial cells and downregulate proangiogenic gene expression, indicating that DHA may exert antiangiogenic effect via regulation of miRNA signature in exosomes.^[^ ^242^ ^]^ Leukemia stem cells (LSCs) are responsible for chemotherapy resistance and relapse of AML. Peng et al. demonstrate that the low level of miR‐34c‐5p in LSCs is associated with the adverse prognosis and poor responses to therapy in AML patients. MiR‐34c‐5p targets RAB27B, a molecule that promotes exosome shedding, to inhibit exosome‐mediated transfer and increase its intracellular level, thus inducing LSC senescence.^[^ ^243^ ^]^ The study from Chen et al. suggests that ovatodiolide, a bioactive ingredient of Anisomeles indica, decreases the expression of miR‐21‐5p in EVs from OSCC stem cells, which resensitizes them to cisplatin and suppresses cancer progression by inhibiting CSC self‐renewal and the transformation of normal fibroblasts to CAFs.^[^ ^244^ ^]^ LncARSR transferred by exosomes promotes sunitinib resistance in RCC cells. Therefore, treatment of sunitinib‐resistant RCC with locked nucleic acids targeting lncARSR or AXL/c‐MET inhibitors restore the responsiveness to sunitinib.^[^ ^120^ ^]^

Inhibiting the internalization of exosomes by target cells may be an additional tool to overcome cancer drug resistance. Berenguer et al. have discovered a novel mechanism for EV uptake, in which the chemokine receptor CCR8 on cells interacts with glycans on the surface of EVs as well as the soluble ligand CCL18. Based on this, they demonstrate that peptide MC148 and small molecule inhibitor R243, two inhibitors for CCR8, could inhibit EV uptake by GBM cells and resensitize them to TMZ.^[^ ^245^ ^]^ EVs from bone marrow stromal cells are internalized by MM cells and reduce their chemosensitivity to bortezomib. Clathrin‐ and caveolin‐dependent endocytosis and macropinocytosis are the main ways of EV‐mediated communication between bone marrow stromal cells and MM cells. Chemical endocytosis inhibitors significantly reduce MM cell internalization of EVs from bone marrow stromal cells and attenuate EV‐mediated chemoresistance to bortezomib, thereby enhancing its anti‐MM effects.^[^ ^246^ ^]^

#### 7.1.2 Targeting EVs to Elicit Anticancer Immune Response to Overcome Cancer Drug Resistance

**Figure 6:**
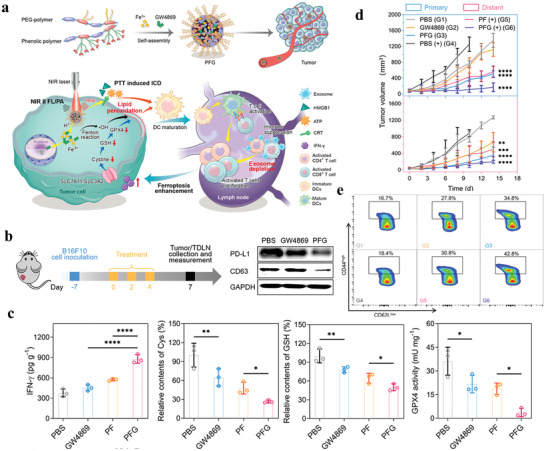
Phototheranostic metal–phenolic networks act with antiexosomal PD‐L1 to enhance ferroptosis for synergistic immunotherapy. a) Schematic illustration of PFG MPNs for phototheranostic effect, relief of exosomal immunosuppression, ferroptosis enhancement, and immune stimulation. b) Scheme of in vivo experiments and western blot analysis of exosomal PD‐L1 and CD63 from tumor tissues after treatment. c) IFN‐*γ* level, relative continents of cystine (Cys)/GSH and GPX4 activity in tumor after treatment. d) Tumor volume of primary and distant tumor. e) Flow cytometric analysis of memory T cells (CD44^high^CD62^low^, gating on CD3^+^CD8^+^ T cells) in the spleen. Reproduced with permission.^[^ ^249^ ^]^ Copyright 2022, American Chemical Society.

PD‐L1/PD‐1 antibodies can reactivate anticancer immune responses, thereby inhibiting cancer progression. Previous studies suggest that PD‐L1 is present on cancer‐derived exosomes. Poggio et al. demonstrate that exosomal PD‐L1 is a target for overcoming cancer cell resistance to antibody immunotherapy. Cancer cells derived exosomal PD‐L1 inhibits T‐cell activation in the draining lymph nodes. However, cancer growth is inhibited when PD‐L1 is removed from exosomes, even in models resistant to anti‐PD‐L1 antibodies. Exposure to exosomal PD‐L1‐depleted cancer cells impedes the growth of wild‐type cancer cells injected at a distant site concurrently or months later. Anti‐PD‐L1 antibodies act synergistically with exosomal PD‐L1 blockade to inhibit cancer growth.^[^ ^247^ ^]^ Lee et al. have recently proposed that macitentan could inhibit the production of EV PD‐L1 in breast cancer cells by targeting the endothelin receptor A, thus improving antitumor immune responses and the therapeutic efficacy of anti‐PD‐L1 antibody by decreasing the binding of PD‐1‐producing T cells to the EV PD‐L1.^[^ ^248^ ^]^ Xie et al. have used the phototheranostic metal–phenolic networks to deliver ferroptosis inducer (Fe^3+^) and exosome inhibitor (GW4869), which elicits a combined photothermal therapy with exosome‐based immunotherapy, revitalizing T cells by antagonizing exosomal PD‐L1‐mediated inhibition and enhances ferroptosis of cancer cells to evoke potent antitumor immunity in melanoma (**Figure** **6**).^[^ ^249^ ^]^

EVs can also be used as an adjuvant for anticancer vaccines to overcome drug resistance. Cheng et al. demonstrate that exosomes from M1 macrophages migrate toward lymph nodes after injection and induce a proinflammatory immune response. Combining M1 exosomes with lipid calcium phosphate nanoencapsulated Trp2 anticancer vaccine can induce a stronger T cell immune response and more efficient antigen‐specific killing than a single anticancer vaccine.^[^ ^250^ ^]^

### 7.2 EVs as Drug Delivery Vehicles for Overcoming Cancer Drug Resistance

Successful cancer treatment depends on the specific and effective delivery of therapeutic drugs to cancer.^[^ ^251^ ^]^ At present, the most commonly used drug delivery vehicles include liposomes, polymer nanoparticles, and phospholipid membrane vesicles.^[^ ^21^, ^252^ ^]^ However, an ideal vehicle that can evade host immune clearance and have long circulation time, high stability, as well as low toxicity remains elusive.^[^ ^253^ ^]^ The biocompatibility and biosafety of polymer nanoparticles remain a concern.^[^ ^254^ ^]^ Phospholipid membrane vesicles, such as EVs, have high loading efficiency and specificity to target cells, thus providing an ideal drug delivery vehicle for overcoming cancer drug resistance.

#### 7.2.1 EV Delivery of Proteins for Overcoming Cancer Drug Resistance

Survivin is an inhibitor of apoptosis protein and is overexpressed in cancer cells. Aspe et al. have used exosomes to deliver Survivin‐T34A, a dominant‐negative mutant of survivin that induces caspase activation and cell apoptosis by dissociating caspase‐9/survivin protein complex.^[^ ^255^ ^]^ Exosomes collected from engineered cells contain Survivin‐T34A and significantly increase GEM‐induced apoptotic cell death, suggesting that the delivery of Survivin‐T34A by exosomes enhances GEM sensitivity in pancreatic adenocarcinoma.

TRAIL is a cancer‐specific proapoptotic protein and the soluble recombinant form of the protein (rTRAIL) has been widely tested in the clinical trials;^[^ ^256^ ^]^ however, the low bioavailability has hindered its clinical application.^[^ ^257^ ^]^ Previous studies demonstrate that EV‐mediated delivery of TRAIL (EV‐T) in a membrane‐bound form could more efficiently induce cell apoptosis. More importantly, EV‐T induces significant apoptosis in TRAIL‐resistant cancer cells, indicating that EV‐TRAIL can replace rTRAIL for cancer treatment to obtain better therapeutic effects.^[^ ^258^ ^]^ The combination of EV‐T with other drug such as SCH727965 (dinaciclib), an effective CDK inhibitor, has been tested in sensitizing the pathway response of cancer cells. Dinaciclib drastically enhances the killing effects of EV‐T on cancer cells that highly express death receptor 5. Combining low‐dose EV‐T with dinaciclib leads to complete cancer regression in mouse models. These observations indicate that EV‐T combined with dinaciclib is a potentially effective approach to improve cancer therapy efficacy.^[^ ^259^ ^]^

#### 7.2.2 EV Delivery of siRNA for Overcoming Cancer Drug Resistance

The key challenge for clinical use of siRNAs is its stability and efficacy, which is tightly linked to their specific delivery to malignant cells. EV‐mediated delivery of siRNAs induces efficient gene silencing in the recipient cells.^[^ ^260^ ^]^ Bellavia et al. have proposed a novel approach to deliver functional BCR‐ABL siRNA by exosomes to overcome CML resistance to imatinib.^[^ ^261^ ^]^ Acquired resistance to sorafenib is a common phenomenon in HCC patients.^[^ ^262^ ^]^ Li et al. have loaded siRNAs that target antiapoptotic proteins into exosomes and anchored these exosomes with EGFR ligands via cholesterol. Cytoplasmic delivery of siRNA overcomes the problem of endosomal capture, leading to effective gene knockdown, chemotherapy sensitization, and cancer regression.^[^ ^263^ ^]^ Previous studies suggest that the level of GRP78 is higher in sorafenib‐resistant cancer cells than sorafenib‐sensitive cells. Li et al. have utilized BMSCs to deliver siGRP78 and revealed that exosomal delivery of siGRP78 enhances the sensitivity of drug‐resistant HCC cells to sorafenib.^[^ ^264^ ^]^ In addition, exosome‐mediated delivery of siRNA efficiently silences circRNA‐SORE in HCC cells, which promotes PRP19‐mediated degradation of YBX1 and significantly increases sorafenib‐induced apoptosis.^[^ ^142^ ^]^

In drug‐resistant CRCs, exosomal delivery of ciRS‐122‐targeting siRNA blocks the ciRS‐122/miR‐122/PKM2 axis, inhibits glycolysis, and increases the response to oxaliplatin.^[^ ^142^ ^]^ Similarly, exosome‐mediated delivery of si‐c‐Met significantly reverses cisplatin resistance in gastric cancer.^[^ ^265^ ^]^ Metastasis‐associated protein 1 (MTA1) is closely related to cancer resistance to chemotherapy and radiotherapy.^[^ ^266^ ^]^ SiMTA1‐loaded exosomes enhance the therapeutic effect of GEM on Luminal‐b breast cancer by inhibiting HIF‐*α* and autophagy.^[^ ^267^ ^]^ Carnitine palmitoyltransferase 1A (CPT1A) is an important enzyme of fatty acid oxidation (FAO) and plays a crucial role in cancer drug resistance. CPT1A siRNA‐loaded and iRGD‐modified exosomes efficiently targets CRCs and inhibit the expression of CPT1A and its downstream gene FAO, reversing their resistance to oxaliplatin,^[^ ^268^ ^]^ which suggests that EV‐delivered siRNAs could efficiently target drug‐resistant cancer cells and restores their therapy sensitivity.

#### 7.2.3 EV Delivery of miRNAs or miRNA Inhibitors for Overcoming Cancer Drug Resistance

Since miRNA can be stably encapsulated in EVs, it is logical to use EVs to deliver therapeutic miRNA to target drug‐resistant genes in cancer cells. Previous studies suggest that EVs loaded with miRNAs targeting cancer‐promoting genes could inhibit cancer growth and reverse cancer drug resistance. For example, miR‐567 expression is decreased in trastuzumab‐resistant breast cancer patients and miR‐567 overexpression by exosome‐mediated delivery reverses therapy resistance by inhibiting autophagy‐related 5.^[^ ^269^ ^]^ Loss of miR‐151a promotes the acquisition of TMZ resistance in GBM. Restoration of miR‐151a in TMZ‐resistant cells through exosomal delivery inhibits XRCC4‐mediated DNA repair and increases therapy sensitivity.^[^ ^270^ ^]^ Exosomal delivery of miR‐146a increases ovarian cancer cell sensitivity to docetaxel and PTX by regulating LAMC2/PI3K/Akt axis.^[^ ^271^ ^]^ Exosome‐delivered miR‐122 increases the sensitivity of HCC cells to soferanib by downregulating multiple resistance‐related genes and inducing apoptosis and cell cycle arrest.^[^ ^272^ ^]^

The abnormal structure of cancer blood vessels affects the sensitivity of chemotherapy drugs. Interestingly, Zhao et al. suggest that miR‐484‐loaded and RGD‐modified exosomes could induce vascular normalization through the inhibition of VEGF‐A expression, thereby increasing the accumulation of chemotherapeutic drugs and enhancing cancer sensitivity to chemotherapeutic drugs.^[^ ^273^ ^]^

Exosomes could also deliver miRNA inhibitor to reverse chemoresistance. For instance, the level of miR‐214 is higher in cisplatin‐resistant gastric cancer cells than their parental cells. Exosome‐mediated delivery of miR‐214 inhibitor reverses chemoresistance and inhibits cancer growth.^[^ ^274^ ^]^ Similarly, the work from our group shows that the level of miR‐374a‐5p is elevated in the serum of patients with gastric cancer and its upregulation indicates a poor prognosis. Exosome‐mediated delivery of miR‐374a‐5p inhibitor increases Neurod1 expression, promotes cell apoptosis, and inhibits oxaliplatin resistance.^[^ ^275^ ^]^ In cisplatin‐resistant OSCC, miR‐155 inhibitor‐loaded exosomes reverse OSCC chemoresistance by upregulating FOXO3a and inhibiting drug efflux transporter protein expression.^[^ ^276^ ^]^ Moreover, previous studies suggest that miR‐21 induces 5‐FU resistance in colorectal cancer through the downregulation of hMSH2 (human DNA MutS homolog 2).^[^ ^277^ ^]^ Based on this, Liang et al. have proposed an approach by simultaneously delivering miR‐21 inhibitor and 5‐FU to HER2^+^ cancer cells via exosomes, which apparently enhances their sensitivity to 5‐FU.^[^ ^278^ ^]^ Similarly, Bose et al. suggest that cancer cell‐derived EVs loaded with miR‐21 inhibitor can block the function of endogenous oncogenic miR‐21 and significantly reduce the resistance of breast cancer cells to DOX.^[^ ^279^ ^]^

#### 7.2.4 EV Delivery of lncRNAs or circRNAs for Overcoming Cancer Drug Resistance

Oxaliplatin resistance is inevitable in most patients with metastatic CRC. A recent study suggests that lncRNA PGM5 antisense RNA1 (PGM5AS1) inhibits the acquired oxaliplatin tolerance of CRCs. Intriguingly, coencapsulation of oxaliplatin and PGM5AS1 in exosomes could efficiently reverse oxaliplatin resistance in CRCs.^[^ ^280^ ^]^ CircRNA FBXW7 (circ‐FBXW7) is downregulated in oxaliplatin‐resistant CRC patients. Exosome‐mediated delivery of circ‐FBXW7 binds to miR‐128‐3p to induce CRC sensitivity to oxaliplatin and inhibit oxaliplatin outflow, which provides a promising strategy for treating oxaliplatin‐resistant CRC.^[^ ^281^ ^]^

#### 7.2.5 EV Delivery of Drugs for Overcoming Cancer Drug Resistance

Most chemotherapeutic drugs have low solubility. Thus, suitable delivery vehicles, such as liposomes and polymer nanoparticles, are needed for systematic administration. Unfortunately, many of these synthetic drug delivery vehicles have serious side effect, which limits their clinical use. By contrast, EVs can efficiently encapsulate chemotherapeutic drugs and facilitate their accumulation in cancer cells and cellular internalization.^[^ ^282^ ^]^ Kim et al. have evaluated the effect of exosome‐based delivery platform for chemotherapeutic drug PTX on the treatment of MDR cancer. Exosomes released from macrophages are loaded with PTX by sonication and the obtained formulation (exoPTX) could not only increase its solubility, but also overcome P‐gp‐mediated drug resistance. Compared with free PTX, exosome‐mediated delivery shows a high loading of PTX and sustained release into the resistant cancer cells to achieve increased cytotoxic effect.^[^ ^283^ ^]^

**Figure 7:**
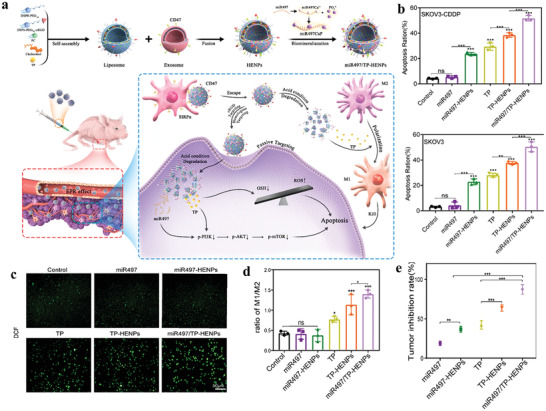
Exosome‐liposome hybrid nanoparticle codelivery of TP and miR497 conspicuously overcomes chemoresistant ovarian cancer. a) Diagram of the formative process and mechanism of action of miR497/TP‐HENPs. b) Quantitative percentages of apoptotic cells in SKOV3‐CDDP and SKOV3 cells. c) ROS in SKOV3‐CDDP cells. d) The ratio of M1/M2 subtype macrophages treated with different groups in vitro. e) The inhibition rate of ovarian cancer treated with various drugs. Reproduced under the terms of a Creative Commons Attribution 4.0 International License.^[^ ^288^ ^]^ Copyright 2022, The Authors. Published by BioMed Central.

In order to overcome the chemotherapy resistance of PDAC, Zhou et al. have loaded PTX and GEM metabolites into exosomes from BMSCs. This platform shows excellent penetration, improved anticancer efficacy, and low systemic toxicity, which provides a prospective approach for targeted PDAC therapy.^[^ ^284^ ^]^ Similarly, cisplatin and PTX loaded in exosomes from macrophages show increased cytotoxicity in drug‐resistant cancer cells.^[^ ^285^ ^]^ Moreover, EVs from autologous prostate cancer cells could be used as an efficient carrier to deliver PTX and enhance the cytotoxic effect of the drug.^[^ ^286^ ^]^ Curcumin is known as an anticancer and anti‐inflammation drug; however, its oral bioavailability is limited due to its hydrophobicity. Exosome‐encapsulated curcumin leads to an increased tissue accumulation of the drug and a consequent decrease in drug resistance as a result of higher uptake, prolonged circulation, and prevention of rapid hepatic degradation.^[^ ^287^ ^]^ Moreover, Li et al. have prepared bioinspired hybrid nanoparticles by fusing CD47‐expressing tumor exosomes with cRGD‐modified liposomes to codeliver chemotherapeutic drug triptolide (TP) and miR‐497, which could target cancer cells to exert a stronger anticancer activity by synergistically inhibiting the activation of PI3K/Akt/mTOR signaling pathway, generating excessive ROS, and repolarizing macrophages from M2 to M1, thus overcoming chemoresistance in ovarian cancer (**Figure** **7**).^[^ ^288^ ^]^ Taken together, EVs provide an effective nanodelivery method for many anticancer drugs, which may help overcome their original limitations in clinical use.

#### 7.2.6 EV Delivery of Functionalized Nanomaterial for Overcoming Cancer Drug Resistance

**Figure 8:**
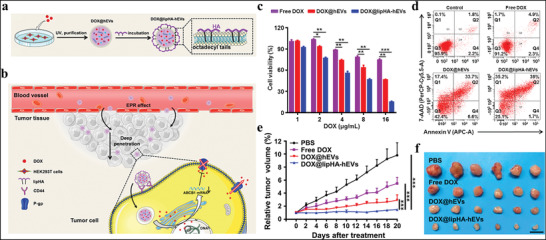
Functional extracellular vesicles engineered with lipid‐grafted hyaluronic acid effectively reverse cancer drug resistance. a) Schematic illustration of the preparation of hEVs membranously engineered with lipid‐grafted HA (lipHA). b) Schematic illustration of lipHA‐hEVs’ delivery route and mechanisms underlying their therapeutic effects against drug resistant tumors. c) Cytotoxicity of free DOX, DOX@hEVs, and DOX@lipHA‐hEVs to MCF7/ADR cells. d) Flow cytometry results of cell apoptosis in MCF7/ADR cells with different treatment. e) Relative tumor volume in the mice receiving indicated treatments. f) Images of the tumors isolated from the tumor‐bearing mice receiving indicated treatments. Reproduced with permission.^[^ ^290^ ^]^ Copyright 2019, Elsevier.

**Figure 9:**
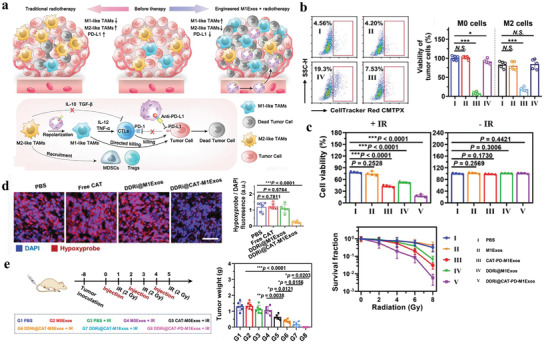
Functional immune cell‐derived exosomes engineered for the trilogy of radiotherapy sensitization. a) Schematic illustration of remodeling macrophages in the tumor suppressive microenvironment. b) The percentage of macrophages undergoing phagocytosis and the overall cytotoxicity of macrophages to tumor cells. I) PBS, II) DDRi@CAT‐PD‐M0Exos, III) DDRi@CAT‐PD‐M1Exos, and IV) DDRi@CAT‐PD‐M2Exos. c) Cell viability and survival fraction of mouse Lewis carcinoma (LLC) cells after treatment with different agents (I–V) and irradiation. d) The degree of tumor hypoxia was examined by the staining with Hypoxyprobe‐1 kit. e) Schedule of in vivo therapy and tumor weight of LLC tumor‐bearing mice. Mice were i.v. injected with different agents (G1–G8) at 5.3 mg kg^−1^ exosomes for three times followed by irradiation (+IR). Reproduced under the terms of the Creative Commons CC‐BY license.^[^ ^293^ ^]^ Copyright 2022, The Authors. Published by Wiley‐VCH GmbH.

Nanotechnology holds great promise for developing new cancer therapeutics with targeted and controlled‐release characteristics. In addition to serving as a delivery tool, EVs can also be modified, engineered, or designed with functional nanomaterials to improve their efficiency, specificity, and safety for cancer therapy.^[^ ^289^ ^]^ For instance, the hyaluronic acid derivative with octadecyl tails (lipHA) are synthesized and attached into the membrane of EVs from HEK293T cells to generate lipHA‐engineered hEVs, which effectively deliver chemotherapeutic drugs to drug‐resistant cancer cells via CD44‐mediated targeting, and simultaneously inhibits the drug efflux by dampening the expression of P‐gp, indicating that cancer‐targeting lipHA‐hEVs could effectively reverse cancer drug resistance (**Figure** **8**).^[^ ^290^ ^]^ Kim et al. have used exosomes to deliver PTX and modified them with aminoethylanisamide‐polyethylene glycol (AA‐PEG), which targets sigma receptors overexpressed in lung cancer cells. The AA‐PEG‐modified, PTX‐loaded exosomes (AA‐PEG‐exoPTX) possess high loading and targeting ability, which greatly improves the therapeutic effect of PTX.^[^ ^291^ ^]^ Thermoperitoneal chemotherapy is currently an effective method for metastatic peritoneal cancer treatment, but this method is limited by drug delivery and rapidly develops drug resistance. Lv et al. have developed a compatible drug delivery system by genetically engineering fibroblasts to produce CD47‐expressing exosomes, then fusing them with thermosensitive liposomes (termed as gETLNPs) to deliver docetaxel. This multimodal combination therapy greatly enhances cancer‐targeting drug delivery ability and effectively overcomes drug resistance.^[^ ^292^ ^]^ Recently, Ma et al. have proposed a concept of using engineered exosomes from M1 macrophage (M1Exos) as sensitizers for effective radiotherapy. They have modified M1Exos with catalases to relieve tumor hypoxia and loaded them with DNA damage repair inhibitor as well as anti‐PD‐L1 nanobody, which together results in an effective remodeling of the immunosuppressive TME and finally an enhanced radiotherapy (**Figure** **9**).^[^ ^293^ ^]^ Wu et al. have used cancer cell‐derived exosomes to deliver POM1 (CD39 antagonist) and metformin (AMPK agonist) for targeted cancer therapy. They demonstrate that this platform increases extracellular level of ATP while decrease that of adenosine, which potentiates the maturation and function of DCs to enhance the cytotoxicity of T cells and NK cells, leading to synergistic antitumor immune responses to suppress cancer progression, metastasis, recurrence, and overcome anti‐PD1 resistance.^[^ ^294^ ^]^

At present, 118 EV‐based clinical studies have been registered on https://clinicaltrials.gov/, of which 25 are related to cancer and these trials could be categorized into EV‐based cancer liquid biopsy and anticancer therapy. About 70% of the existing trials have tested the potential of EVs as biomarkers for cancer diagnosis and prognosis, and the others focus on cancer therapy by taking use of EVs as anticancer drug delivery vehicles or vaccines. Of these studies, 3 are completed, 15 are in actively recruiting, 2 are active but not yet recruited, 2 are not recruiting, and 3 are not clear. A phase I (NCT03608631) clinical trial is enrolling patients with KrasG12D‐mutated metastatic pancreatic cancer and evaluating the efficacy of iExosomes, an engineered exosome‐based delivery platform for Kras‐targeting siRNA. Another phase II clinical trial vaccinates 47 NSCLC patients with a tumor antigen‐loaded DC‐EVs vaccine and assesses patients’ responsiveness to induction chemotherapy (NCT01159288). There is also a phase I clinical trial investigating the ability of plant exosomes to deliver curcumin to normal and colon cancer tissues (NCT01294072). Moreover, several commercial companies are focusing on developing EV‐based anticancer therapeutics, such as exoIL‐12, exoSTING, and exoASO‐STAT6 from Codiak BioSciences, and have achieved encouraging results in preclinical models and clinical trials.^[^ ^295^ ^]^ Up to date, there is no clinical trial that utilizes EVs to overcome cancer drug resistance. Therefore, further studies are needed to investigate EV‐based therapeutics on the reversal of cancer drug resistance.

## 8 Perspective and Future Challenges

Drug resistance is a major obstacle for effective cancer therapy. Recently, EVs have been regarded as a new mode of intercellular communication, and have attracted wide attention in cancer liquid biopsy and cancer therapy as a result of their unique characteristics and critical cellular functions. Herein, we summarized the biological roles of EVs in the emergence and progression of drug resistance and their underlying molecular mechanisms. The specific cargos carried by EVs, such as drug efflux pumps, proteins, mRNAs, and ncRNAs, transmit the trait of resistance from drug‐resistant cells to their sensitive counterparts. Moreover, EVs mediate the interaction between cancer cells and TME cells to confer drug resistance. The identification of these cargos and the elucidation of their mechanisms of action will help clarify the functions of EVs in cancer drug resistance. Therefore, an in‐depth understanding of the relationship between EVs and drug resistance will promote the development of more effective approaches to dynamically monitor therapy response and restore the sensitivity of cancer cells to the therapeutics.^[^ ^253^ ^]^

Previous studies has yielded some encouraging results but there are still many hurdles that need to be addressed before the clinical translation of EVs in the monitoring of therapy response and treatment of drug‐resistant cancer. Although EVs show great potential in predicting the efficacy of cancer treatments, an efficient isolation method for large‐scale purification of homogeneous EVs populations has not been established. Current methods for EVs enrichment, isolation, and analysis have limitations, such as a lack of sensitivity, specificity, and reproducibility. High cost and unsatisfactory separation efficiency are the main problems currently limiting the production of large‐scale EVs. Therefore, more convenient, stable, streamlined, and cost‐effective assay platforms are needed to utilize EVs as indicators of therapy resistance. Furthermore, the biological properties of EVs secreted by different cells vary, and this heterogeneity also affects the cancer specificity of the identified EVs markers.^[^ ^296^ ^]^ How to efficiently and rapidly isolate EVs from complex blood and body fluid samples to achieve sensitive, specific and accurate detection is crucial for the clinical translation of EVs. The natural properties of EVs make them ideal tools for drug delivery. EVs show great potential in overcoming cancer drug resistance by delivering siRNA, miRNAs, drugs, and functionalized nanomaterials. However, their clinical applications are still in their infancy. There are many practical problems to be solved in the process of clinical application and translation of EVs for overcoming drug resistance. For example, the route of administration, biodistribution, sustained biological effects, potential toxicity, and effective dose of EVs‐based drug delivery vehicles, as well as drug modification/loading strategies for EVs and finally, massive production and postproduction quality detection.^[^ ^297^ ^]^ Despite their scientific robustness, most experimental evidence for EVs and drug resistance comes from preclinical models with limited patient sample numbers. Although EVs have been used to treat cancer patients since early 2000s, but so far, no standard techniques for clinically graded production and quality control of EV‐based therapeutics have been established. Therefore, establishing clinical trials that meet all necessary criteria is one of the great challenges in EVs‐based cancer therapy.

In conclusion, with the increasingly active study on EVs and cancer as well as the rapid development of advanced technology for EV detection and engineering, the resolution of these key issues will make EVs a new strategy for predicting cancer therapy response and improving cancer therapy efficacy.

## Conflict of Interest

The authors declare no conflict of interest.
